# Antileishmanial Anthracene Endoperoxides: Efficacy In Vitro, Mechanisms and Structure-Activity Relationships

**DOI:** 10.3390/molecules27206846

**Published:** 2022-10-13

**Authors:** Laura Machin, Martin Piontek, Sara Todhe, Katrin Staniek, Lianet Monzote, Werner Fudickar, Torsten Linker, Lars Gille

**Affiliations:** 1 Department of Biomedical Sciences, Institute of Pharmacology and Toxicology, University of Veterinary Medicine, 1210 Vienna, Austria; 2 Pharmacy Department, Institute of Pharmacy and Food Sciences, University of Havana, Havana 13600, Cuba; 3 Parasitology Department, Institute of Tropical Medicine “Pedro Kouri”, Havana 11400, Cuba; 4 Department of Organic Chemistry, Institute of Chemistry, University of Potsdam, 14476 Potsdam, Germany

**Keywords:** endoperoxides, *Leishmania*, radicals, iron, anthracene, electron paramagnetic resonance

## Abstract

Leishmaniasis is a vector-borne disease caused by protozoal *Leishmania* parasites. Previous studies have shown that endoperoxides (EP) can selectively kill *Leishmania* in host cells. Therefore, we studied in this work a set of new anthracene-derived EP (AcEP) together with their non-endoperoxidic analogs in model systems of *Leishmania tarentolae* promastigotes (LtP) and J774 macrophages for their antileishmanial activity and selectivity. The mechanism of effective compounds was explored by studying their reaction with iron (II) in chemical systems and in *Leishmania*. The correlation of structural parameters with activity demonstrated that in this compound set, active compounds had a LogP_OW_ larger than 3.5 and a polar surface area smaller than 100 Å^2^. The most effective compounds (IC_50_ in LtP < 2 µM) with the highest selectivity (SI > 30) were pyridyl-/tert-butyl-substituted AcEP. Interestingly, also their analogs demonstrated activity and selectivity. In mechanistic studies, it was shown that EP were activated by iron in chemical systems and in LtP due to their EP group. However, the molecular structure beyond the EP group significantly contributed to their differential mitochondrial inhibition in *Leishmania*. The identified compound pairs are a good starting point for subsequent experiments in pathogenic *Leishmania* in vitro and in animal models.

## 1. Introduction

Leishmaniasis is a vector-borne parasitic disease preferentially occurring in tropical and subtropical countries [1]. The disease is caused by infection of mammals with protozoal parasites from the genus *Leishmania*. Due to a variety of *Leishmania* species clinical manifestations of the disease in humans can range from cutaneous over mucocutaneous to visceral forms, which can even lead to death [2,3]. The epidemiological situation for this disease is complicated on the one hand by its zoonotic occurrence and by altered distribution of the vector due to climate change [4]. According to the World Health Organization (WHO), leishmaniasis is one of the seven most important tropical diseases and represents a serious threat to global health [2]. Leishmaniasis management requires integrated and multidisciplinary strategies. However, due to the absence of a vaccine for humans, drug therapy is the only available alternative to leishmaniasis control [5].

WHO recommends leishmaniasis treatment based on causative species, geographical area, and clinical symptoms [6]. Current treatment approaches are subdivided into local therapy, including topical and intralesional administrations, and systemic therapy with drugs, such as antimonials, formulations of amphotericin B, miltefosine and paromomycin [7,8].

Current therapeutic approaches still rely heavily on chemotherapy with compounds having also often disadvantages, such as cost, availability, side effects, resistance development, and pharmacokinetic limitations in different clinical forms [9]. This urges the demand for new safe and efficient antileishmanial therapies. Furthermore, also problems in the drug discovery process itself, due to the intracellular presence of *Leishmania* in macrophages and various in vitro models, complicate leishmaniasis drug development [10,11]. Organic endoperoxides (EP) of natural origin, such as artemisinin and ascaridole, have demonstrated antiprotozoal effects in vivo and in vitro against *Plasmodium* and *Leishmania* [12,13,14,15]. In *Leishmania* the prerequisite for the efficiency of EP is the abundance of iron and heme in these protozoal organisms as well as their limited protection against oxidative stress [16,17]. Furthermore, the benefit of EP for the treatment of intracellular *Leishmania* in macrophages is their slow degradation in the mammalian host [18,19]. In addition to natural EP, structures based on trioxolanes have also been tested for their activity against *Leishmania* [20]. Anthracene endoperoxides (AcEP) originate from technical applications for UV photolithography [21], although their biological and pharmacological potential was less explored so far [22].

In a previous study, we demonstrated that simple substituted AcEP possess antileishmanial activity in vitro, with, however, limited selectivity for parasites over macrophages [23]. Based on these findings, we decided to study a larger variety of AcEP with more different substitution patterns. The study was carried out to reveal the antileishmanial activity of AcEP including their selectivity, the mechanism of activation, and the pharmacological consequences in *Leishmania*, such as mitochondrial inhibition and superoxide formation. Based on structure–activity relationships we identified structural features supporting antileishmanial activity and selectivity as a guide for future synthesis and selection of compounds for in vivo activity tests.

## 2. Results

### 2.1. Compounds and Structural Properties

**Figure 1:**
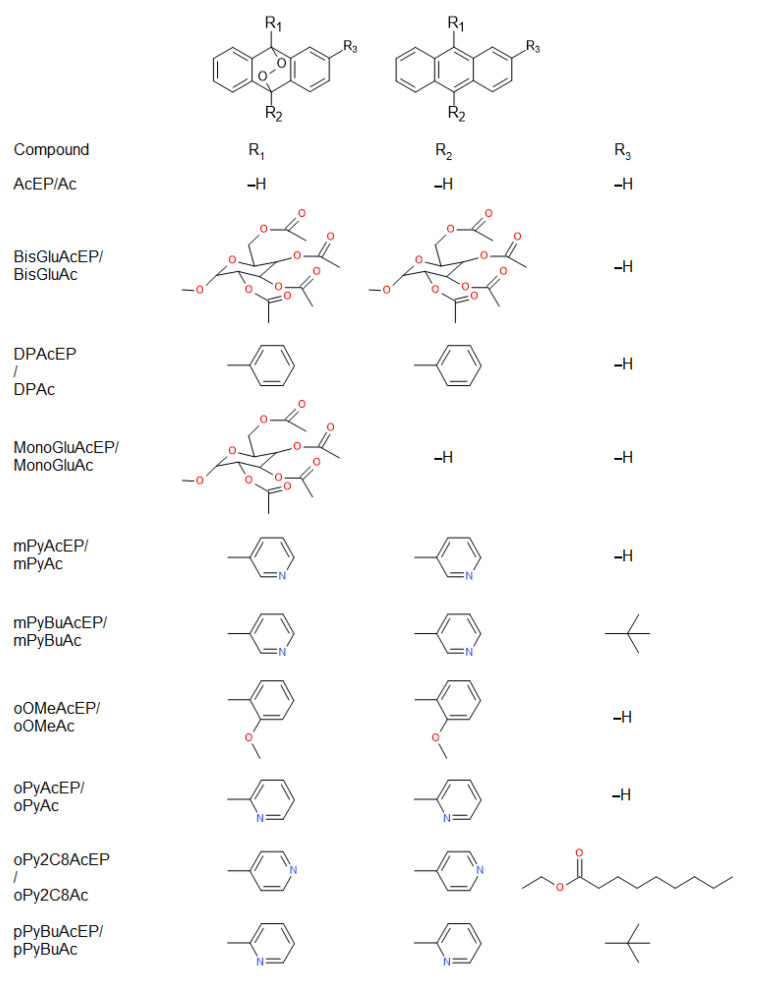
Compound pairs of anthracene endoperoxides (AcEP) and their non-endoperoxidic analogs (Ac) included in the study of antileishmanial activity. The figure shows principal structures of AcEP/Ac (top) followed by the compound abbreviations and the respective substituents (bottom).

**Figure 2:**
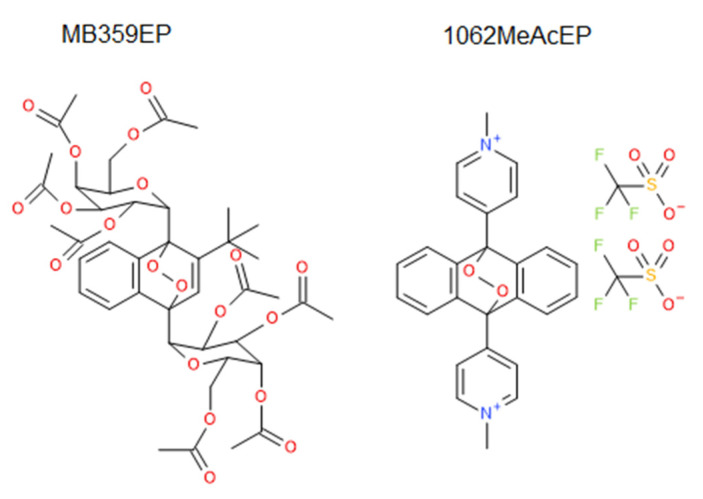
Endoperoxides included in the study without non-endoperoxidic analogs.

For this study, EP based on the anthracene moiety with different substituents were used (Figure 1). Furthermore, the non-endoperoxidic analogs (Ac) of these EP were included to dissect effects caused by the EP group from effects caused by the other structural elements of the molecules (Figure 1). In addition, two EP without non-endoperoxidic analog were included due to their unique substituent structure (Figure 2).

Based on the structural formulas the bioavailability parameters of the selected compounds were calculated using the software BIOVIA Draw (Table 1). These data demonstrate that compounds over a wide range of molecular weight, partition coefficient (LogP_OW_), polar surface area (PSA), different numbers of hydrogen bond acceptors, and stereo centers were included. None of the molecules exhibited hydrogen bond donor centers.

### 2.2. Influence on LtP and J774 Viability

The influence of AcEP and their analogs (Ac) on the viability of cells was tested in *Leishmania tarentolae* promastigotes (LtP) and J774 macrophages (Table 2). Based on the changes of viability of the respective cell lines at different compound concentrations, IC_50_ values were calculated. In Table 2, the IC_50_ values in both cell lines were compared for the individual compounds. From the ratio of IC_50_ values in J774 macrophages to IC_50_ values in LtP, an in vitro selectivity was calculated. IC_50_ values for *Leishmania* show that some compounds, including some EP, did not kill LtP in vitro at 200 µM. On the other hand, most effective compounds exhibited IC_50_ values for *Leishmania* in the submicromolar range. Surprisingly, also some non-endoperoxidic analogs (Ac) demonstrated low IC_50_ values and a high antileishmanial activity. The toxicity of the compounds in J774 macrophages strongly varies. While some compounds have IC_50_ values far higher than 100 µM, a few show IC_50_ values in the low micromolar range for J774 cells. The most selective and effective compounds in these test systems, which have a higher selectivity than the reference compound pentamidine (Pen), are bipyridyl-substituted derivatives of tert-butyl substituted Ac and the corresponding EP.

### 2.3. Activation of AcEP by Iron in Chemical Systems

**Figure 3:**
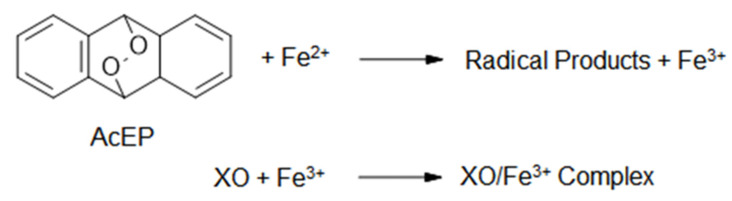
Reaction of AcEP with iron (II) under formation of AcEP radicals and iron (III), which subsequently forms a complex with xylenol orange (XO).

In subsequent experiments, we explored the activation of EP and possible cellular consequences of these compounds in *Leishmania*. In a chemical system, we tested how fast EP react with iron (II) and, therefore, lead to the activation of these compounds. Resulting iron (III) was detected as complex with xylenol orange (XO, Figure 3). The reaction rate of the EP stretched across two magnitudes of orders (Table 3). On the first view, there is no direct link between the activation rates and the efficiency of the compounds against *Leishmania*. In contrast, most efficient compounds in *Leishmania*, such as mPyBuAcEP and pPyBuAcEP, exhibited a rather slow activation by iron (II). For comparison purposes, we displayed in Table 3 also the ratio of IC_50_ values of non-EP in LtP versus the IC_50_ values of their respective EP.

**Figure 4:**
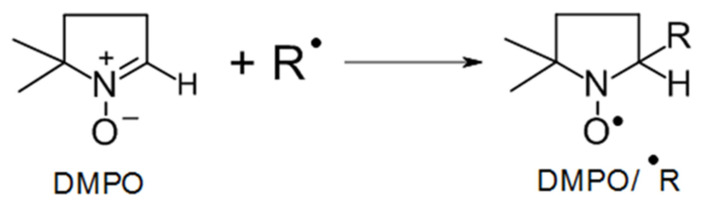
Reaction of DMPO with a radical (R^•^) under the formation of a spin adduct DMPO/^•^R, which then can be detected by EPR spectroscopy.

**Figure 5:**
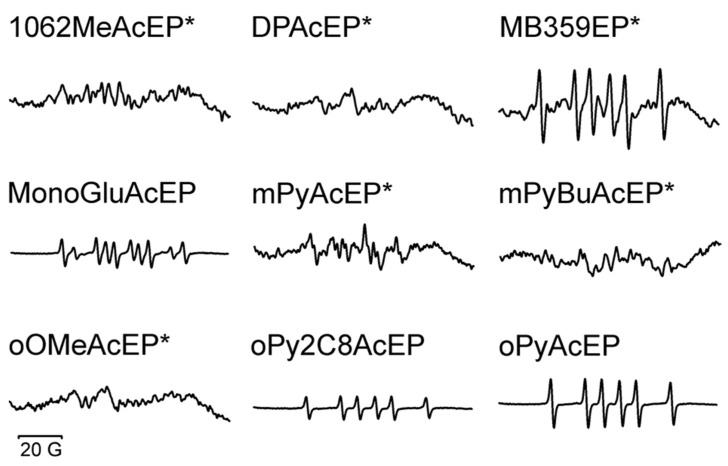
EPR spectra recorded after 5 min incubation of 500 µM AcEP with 500 µM FeSO_4_ at room temperature in H_2_O with 40 mM DMPO. * EPR spectra recorded after 10 min incubation of 4 mM AcEP with 1 mM FeSO_4_ at 50 °C in H_2_O/acetonitrile (8:2) with 40 mM DMPO. Total scan range was 100 G. The figure shows representative spectra of at least three individual experiments.

As demonstrated in the XO assay, all tested EP reacted with iron (II). Electron transfer to the EP group should result in the formation of oxygen-centered radicals, which then can further stabilize by rearrangement to carbon-centered radicals. Electron paramagnetic resonance (EPR) spectroscopy/spin trapping using 5,5-dimethyl-1-pyrroline N-oxide (DMPO) as spin trap compound (Figure 4) was used to study this possibility in selected AcEP. The resulting EPR spectra are shown in Figure 5. As can be seen, the intensity of the obtained spin adducts was considerably different from compound to compound. Clear identifiable splitting patterns were obtained for MB359EP, MonoGluAcEP, oPy2C8AcEP, and oPyAcEP. Spectral simulations yielded coupling constants summarized in Table 4. These data revealed the presence of DMPO spin adducts with carbon-centered radicals in all spectra with high signal intensity.

### 2.4. Activation of AcEP by Cycloreversion

**Figure 6:**
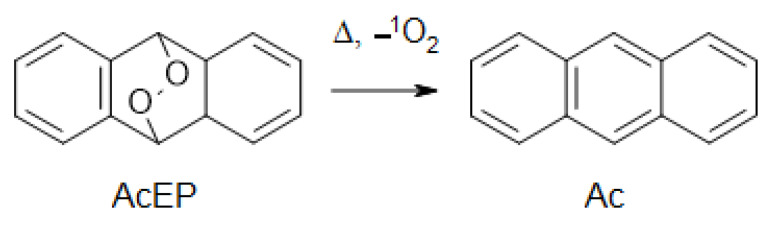
Cycloreversion of AcEP to Ac and singlet oxygen.

Besides the possibility of AcEP activation by transition metal reduction in cells, compounds can also undergo thermal decomposition by cycloreversion, reforming the parent Ac compound and singlet oxygen (Figure 6).

**Figure 7:**
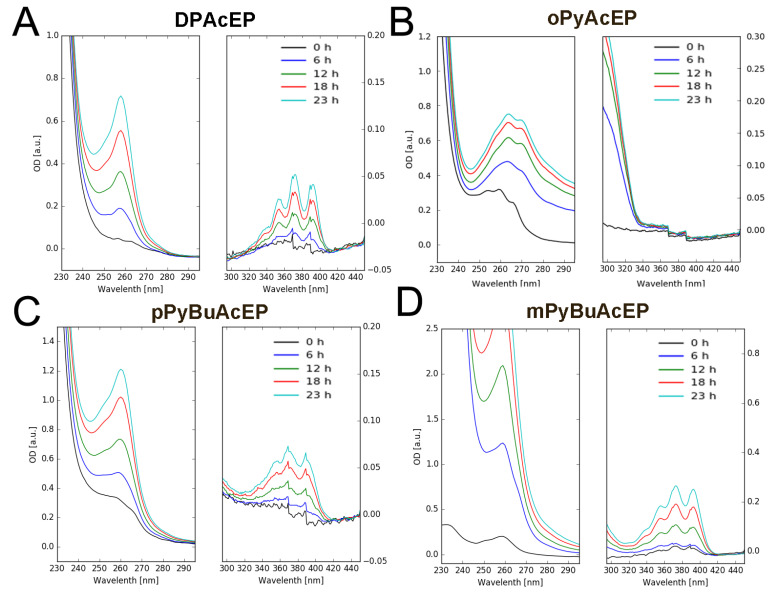
UV spectra of thermal decomposition of AcEP. Spectra show the spectral changes of AcEP (40 µM) in EtOH at 70 °C over up to 23 h. Reformation of the corresponding Ac spectral pattern indicates cycloreversion of the AcEP. (**A**) DPAcEP, (**B**) oPyAcEP, (**C**) pPyBuAcEP and (**D**) mPyBuAcEP. Spectra of non-endoperoxidic Ac analogs (40 µM) are shown in Figure S1 (Supplement). Spectra are typical examples of 2–3 replicates.

Therefore, we tested for most effective EP whether they can undergo cycloreversion at 70 °C in ethanol (EtOH) over 24 h. To determine EP decomposition, we recorded at first the UV spectra of the parent Ac compound and then observed the spectral changes of the corresponding EP during incubation in EtOH (Figure 7). As can be seen from the series of recorded spectra, DPAcEP, pPyBuAcEP, and mPyBuAcEP showed a clear reformation of the parent Ac compound over time. However, oPyAcEP did not undergo conversion to the parent Ac compound (Figure 7 and Figure S1). Spectral changes in these compounds rather indicate other decomposition pathways. These experiments suggest that cycloreversion may also take place in the cellular environment, though with a rather low reaction rate.

### 2.5. Role of Thiols and Iron in AcEP Actions in LtP

**Figure 8:**
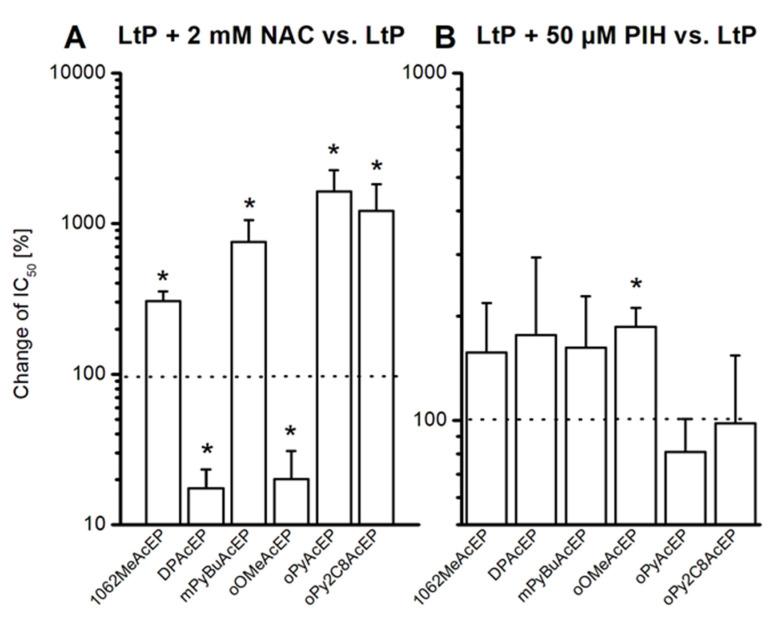
Change of IC_50_ values (%) of EP in LtP triggered by the addition of 2 mM NAC (**A**) or 50 µM PIH (**B**). IC_50_ values were determined using resazurin viability assays with 2 × 10^6^ LtP/mL. Data represent mean ± SD of 4–6 individual experiments. * Significant differences to the control (LtP with the respective compound, but without NAC and PIH) at *p* < 0.05.

Based on the findings from chemical test systems, we studied the actual activation of these EP compounds in LtP. For this purpose, we used the established viability assays for *Leishmania* and studied how IC_50_ values were changed by the presence of the typical radical scavenger N-acetyl cysteine (NAC) or the iron chelator for low molecular iron pyridoxal isonicotinoyl hydrazine (PIH). We expressed the changes of the IC_50_ values in the presence of these additional compounds in percent, referring to the original IC_50_ values in LtP set to 100% (Figure 8). As can be seen for most of the tested AcEP, the IC_50_ value increased for up to one order of magnitude in the presence of NAC. Interestingly, DPAcEP and oOMeAcEP did not respond, but rather displayed even lower IC_50_ values. Likewise, in the presence of the iron chelator PIH, the IC_50_ values of most EP in LtP shifted to higher values. However, oPyAcEP and oPy2C8AcEP did not respond in this manner.

### 2.6. Influence on Oxygen Consumption and Superoxide Formation

**Figure 9:**
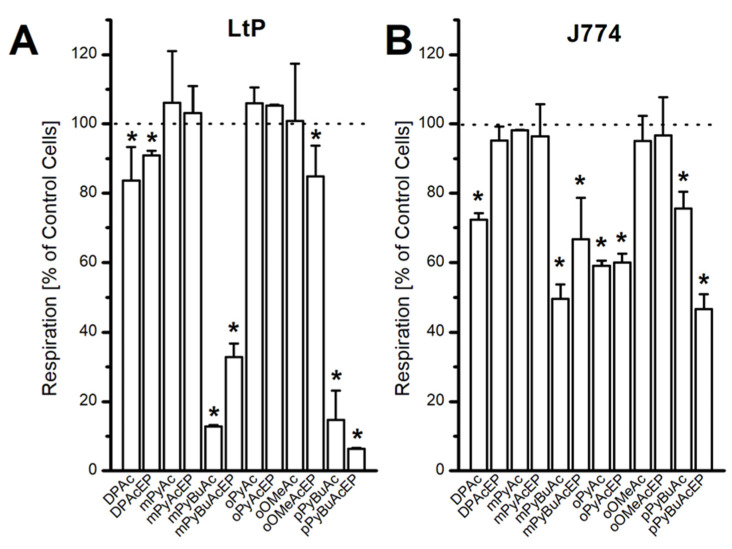
Inhibition of cellular oxygen consumption in LtP and J774 cells in the presence of 200 µM Ac/AcEP compounds. Remaining respiration is expressed as percentage of respiration of control cells (**A**) LtP (140 × 10^6^ cells/mL) or (**B**) J774 (2 × 10^6^ cells/mL). Data represent mean ± SD, *n* = 4. * Significant differences to the respective control (respiration of LtP or J774 without any compound on the same plate, set to 100%) at *p* < 0.05.

The experimental data so far indicate that in more complex EP not only the endoperoxyl group contributes to its pharmacological actions. Therefore, mitochondria present in the parasites but also in the host cells could be either a trigger for activating pharmacological actions of these compounds or a target of EP-derived radicals generated at other sites in the cell. In a subsequent experiment, we tested the influence of AcEP and non-endoperoxidic analogs (Ac) on the mitochondrial oxygen consumption in LtP and J774 macrophages (Figure 9). As can be seen from the resulting data, most EP and analogs do not strongly interfere in mitochondrial respiration at concentrations of 200 µM. However, some derivatives, such as mPyBuAcEP, pPyBuAcEP and their analogs, interfered strongly in the mitochondrial function in LtP at these concentrations, while the effect on mitochondrial respiration in J774 macrophages was more moderate.

**Figure 10:**
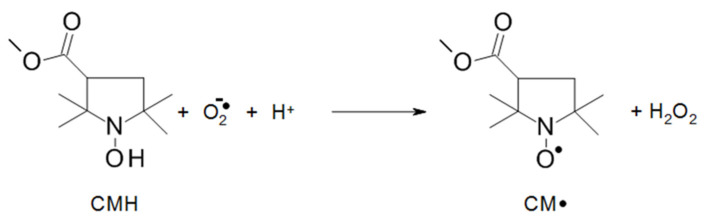
Oxidation of CMH by a superoxide radical under the formation of a nitroxyl radical CM^●^, which then can be detected by EPR spectroscopy.

**Figure 11:**
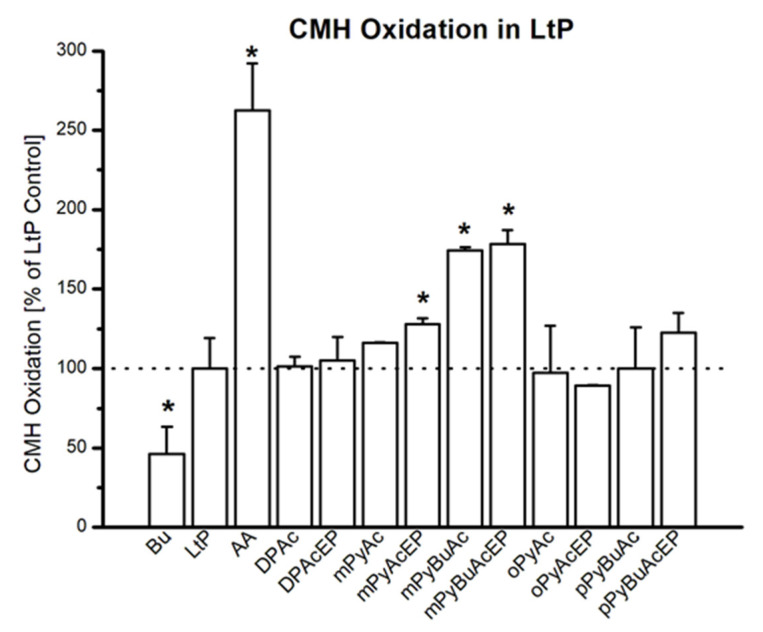
Oxidation rate of CMH by superoxide radicals to CM**^●^** in buffer (Bu) or LtP in the absence and presence of antimycin A (AA, 20 µM) or Ac/AcEP (100 µM) after 20 min incubation at 25 °C. Data represent mean ± SD, *n* = 3 or 7 (Bu, LtP, AA). * Significant differences to the respective control (LtP without any compound) at *p* < 0.05.

Since mitochondrial inhibition can be triggered by secondary damage in the respective cells, for example, by superoxide radicals, we studied the formation of superoxide radicals in LtP by the EPR/hydroxyl-3-methoxycarbonyl-2,2,5,5-tetramethylpyrrolidine hydrochloride (CMH) method (Figure 10). Superoxide formation measured as CMH oxidation in LtP shows that the buffer samples (without LtP) demonstrate about half as much CMH oxidation as LtP. In the presence of antimycin A (AA, an inhibitor of mitochondrial complex III), the superoxide radical formation in LtP is increased by more than 150% (Figure 11). This demonstrates that the test system is able to provide quantitative information about the superoxide radical formation in *Leishmania*. In the presence of AcEP and the non-endoperoxidic analogs (Ac) at concentrations of 100 µM, only few compounds increased superoxide formation significantly. Interestingly, most effective in boosting the superoxide production in LtP were mPyBuAcEP and its non-endoperoxidic analog mPyBuAc. These compounds also inhibited oxygen consumption in LtP and were highly effective and selective against *Leishmania* versus macrophages.

## 3. Discussion

Available classical drugs for the treatment of leishmaniasis have several problems, such as increasing resistance, toxic side effects, requirements of hospitalization, availability and cost. Furthermore, finding new effective antileishmanial agents is complicated by the different clinical manifestations of leishmaniasis and the fact that even for one clinical form of leishmaniasis different species can be responsible (Old world vs. New World species), which exhibit a different drug sensitivity [24]. Irrespective of the later clinical application of drugs against a specific *Leishmania* species, a first step is the identification of active compounds against *Leishmania* in vitro. A simple test system, which allows screening and easy mechanistic experiments, is *Leishmania tarentolae* in their promastigote (LtP) and amastigote forms [25]. They have the advantage of being non-pathogenic and can be grown in large quantities for biochemical experiments. This test system is no replacement for assays with intracellular amastigotes of pathogenic *Leishmania* species, but it is a suitable starting point to preselect possible antileishmanial compounds and can provide valuable mechanistic insights.

The problems with current antileishmanials triggered the search for new drug classes against leishmaniasis. From the benefit of artemisinin derivatives against *Plasmodium* [26], the idea arose whether artemisinins and EP in general can be beneficial against *Leishmania* [15]. However, the physiological situation in erythrocytes parasitized by *Plasmodium* is different from macrophages parasitized by *Leishmania*. In the malaria disease, *Plasmodium* parasites feed on degraded hemoglobin from erythrocyte host cells, which is accompanied by release of toxic heme, making them vulnerable for peroxides. In context to the antimalarial action of artemisinins and related EP, it was shown that cleavage of the peroxyl group by low molecular iron is an important trigger for pharmacological activation of these compounds [12,27].

*Leishmania* parasites acquire low molecular iron, but also heme compounds, from the host macrophage cell [28,29]. This makes them sensitive to peroxides, but their reaction partners are more versatile including low molecular iron and heme compounds. We have shown in the past that the natural endoperoxide ascaridole preferably interacts with low molecular iron in *Leishmania*, generating carbon-centered radicals [16]. On the other hand, artemisinin was demonstrated in *Leishmania* to interact with imported and degraded heme compounds as well [17].

In the later stage of drug development, it is also important that respective EP be preferably activated in the target cells and not during the pharmacokinetic phase of uptake and distribution. Interestingly, it has been shown that trioxolanes exhibit a certain stability in the presence of hemoglobin [19]. In general, a certain chemical stability of EP could also be beneficial in the case of leishmaniasis, since drugs have to accumulate in macrophages and have to escape (at least partially) the host body metabolism.

As antiplasmodial drugs several types of EP have been explored in the past: artemisinins and semi synthetic derivatives, trioxolanes (ozonids) [30,31], and ascaridole [32]. Under hypoxic conditions in mammalian cells, some EP were shown to release O_2_ contributing to cell survival [33]. Against *Leishmania*, similar and other types of EP have been explored for their activity in vitro and in vivo, including: cyclic peroxides from marine organisms [34], ascaridole from *Dysphania ambrosioides* (L.) Mosyakin & Clemants [16,35], ergosterol endoperoxide [36], and artemisinin [14,37]. Although natural EP are highly interesting due to their diversity in structure, they are often difficult to synthesize de novo and, therefore, make systematic variation of structures difficult. Therefore, sets of synthetic EP were developed and tested against *Leishmania* [20,23].

Complex substituted AcEP have been studied as photo switches or photoresists [21]. While photoresists are preferably known from technical applications, such as photolithography for semiconductor production, photo switches can also exert biological functions, e.g., in rhodopsin, and are characterized by conformational changes during photochemical reactions.

Diaryl-alkyl AcEP possess a thermal stability up to 120 °C, which, however, does not exclude potential decomposition over the course of pharmacological applications (several days). Little is known about the biological activity of substituted AcEP. It has been shown that certain pyridinium anthracenes have an affinity to the minor grooves of DNA [22].

In a previous work, we tested four AcEP substituted by methyl groups together with unsubstituted anthracene and anthraquinone as reference compounds [23]. In viability tests performed in LtP, axenic *Leishmania tarentolae* amastigotes and *Leishmania donovani* promastigotes specifically substituted AcEP showed IC_50_ values in the low micromolar range (some < 10 µM). In contrast, reference compounds, such as anthracene and anthraquinone, demonstrated considerably higher IC_50_ values (>100 µM). A major problem of these AcEP was the high cytotoxicity (low IC_50_ values) in J774 macrophages, which represented a model for host macrophages. Therefore, irrespectively whether we compared IC_50_ values in J774 cells to different *Leishmania* models, the selectivity of these AcEP for the parasite was rather low. Nevertheless, this work gave important mechanistic insights. For these simple AcEP, it was shown that they are activated to carbon-centered radicals within cells and that this pathway is important for the pharmacological action. On the other hand, most of these compounds did not primarily target *Leishmania* mitochondria. These results triggered us to study a more diverse set of AcEP substituted with heterocycles, along with their non-endoperoxidic analogs.

The compounds studied in our work consisted preferably of anthracene-derived EP (AcEP) and their parent anthracene analogs (Ac) with different substitution patterns (Figure 1). In addition, two separate acene EP were included: MB359EP and 1062MeAcEP (Figure 2). Besides unsubstituted AcEP, the compounds were substituted by either carbohydrate residues (MonoGluAcEP and BisGluAcEP), phenyl residues (DPAcEP and oOMeAcEP) or pyridyl residues (mPyAcEP, mPyBuAcEP, oPyAcEP, oPy2C8AcEP, and pPyBuAcEP) in 9, 10 position of the anthracene ring (Figure 1). In addition, some of these compounds exhibited an additional substituent at position 2 of the anthracene ring (mPyBuAcEP oPy2C8AcEP, pPyBuAcEP).

The QSAR parameters in Table 1 show that model compounds cover a wide range of molecular weights from about 178 to 903 Da. The predicted lipophilicity ranges from a LogP_OW_ of 1.03 to 8.41. Specifically, carbohydrate-substituted AcEP exhibit a high number of hydrogen bond acceptors, while none of the compounds exhibited any hydrogen bond donor groups. Among substituted AcEP, those with additional *tert*-butyl substitution at the anthracene ring exhibited several stereo chemical centers. The predicted PSA of the compounds ranges from 0 to about 270 Å^2^ and give predictions about the ability of compounds to permeate cellular membranes.

In viability assays, the influence of compounds on LtP and J774 macrophages was tested, and IC_50_ values for cell survival were obtained (Table 2). The reference compound Pen shows a submicromolar IC_50_ value for LtP and an IC_50_ value for macrophages of about 12 µM, resulting in a selectivity index of around 24. IC_50_ values for AcEP and parent compounds range from submicromolar values up to concentrations above 200 µM. This shows that the EP group contributes to their antileishmanial activity and sometimes cytotoxicity but does not exclusively determine the efficiency of the compounds.

**Figure 12:**
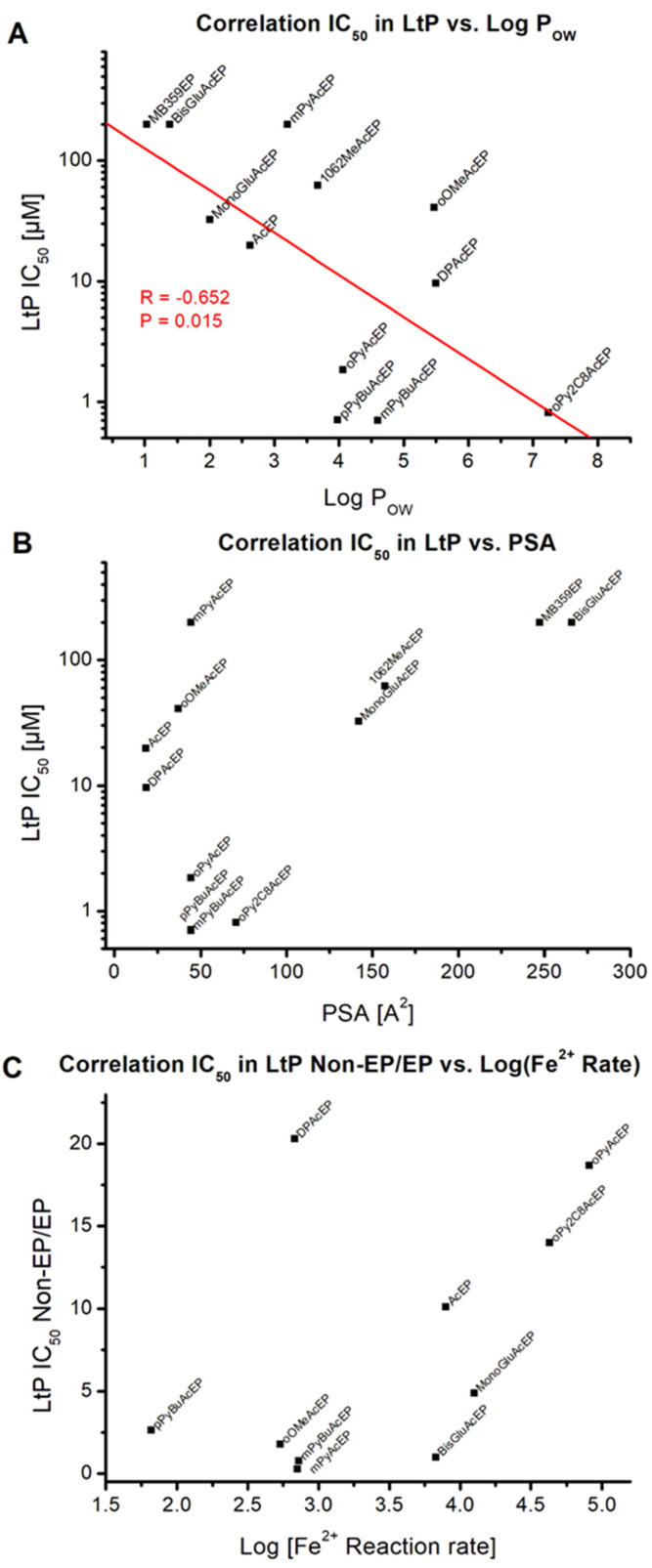
Possible relationships between different properties of AcEP. Correlation of IC_50_ values for EP actions on LtP viability vs. LogP_OW_ (**A**) and PSA (**B**) of compounds. Correlation of the ratio of IC_50_ values of non-EP/EP in LtP versus the reaction rate of EP with Fe^2+^ (**C**).

Least effective were the carbohydrate substituted AcEP and their parent compounds. Among the pyridyl-substituted compounds, some are effective at submicromolar concentrations against LtP, while some of them are also toxic in J774 macrophages. If one correlates the IC_50_ in LtP on a logarithmic scale with LogP_OW_ (Figure 12A), a negative linear correlation is observed, predicting that a higher lipophilicity is often associated with lower IC_50_ values in LtP. This effect matches the observation in whole mammals that lipophilic drugs (high LogP_OW_) have a higher activity in the central nervous system (lower ED_50_) due to their better permeability across the blood–brain barrier [38]. Correlating the IC_50_ values in LtP with the PSA of the molecules, it becomes visible that for PSA values below 100 Å^2^ both active and inactive compounds were observed. However, for compounds with PSA values larger than 100 Å^2^, no active compounds were found (Figure 12B). This fits with the observation for other drugs suggesting that cell permeability of molecules is poor above approximately 140 Å^2^ PSA [39].

Most effective compound pairs with respect to a low IC_50_ value in LtP and a reasonable selectivity for the parasite over J774 cells, in which both compounds show activity were mPyBuAcEP/mPyBuAc and pPyBuAcEP/pPyBuAc. For DPAcEP, which is rather active against LtP, the non-endoperoxidic analog DPAc is rather inactive.

To explore the role of the EP group in AcEP, we studied the reaction rates of the EP with iron (II) in a chemical environment (Figure 3 and Table 3). Resulting formation of iron (III) was detected by complex formation with XO. Reaction rates differed among compounds over several magnitudes. For comparison purposes, the ratio of the IC_50_ values from the Ac analogs to the IC_50_ value of their corresponding AcEP is listed in Table 3. The correlation of this ratio with the logarithm of the reaction rate with iron (II) (Figure 12C) shows that for fast reacting EP the ratios for the IC_50_ values in LtP tend to be very high (with the exception of DPAcEP). Accordingly, for more slowly reacting EP, the IC_50_ values of the AcEP and their analogs become more similar. Thus, for quickly reacting EP, the endoperoxyl group tends to be more important for their pharmacological action.

In a next step, we explored the possible reaction products of EP with iron (II) in a buffer system by EPR spin trapping using DMPO as spin trap (Figure 4). Because of the extremely different reactivity, not for all EP spin adduct signals with a sufficient signal-to-noise ratio could be obtained (Figure 5). For EPR signals with sufficient quality simulation calculations were performed (Table 4). Resulting coupling constants indicate the presence of carbon-centered radicals for MonoGluAcEP, oPy2C8AcEP, oPyAcEP, and MB359EP. For other compounds, the formation of carbon-centered radicals in this buffer system could not be verified by this method. This could mean that this pathway is less important for the respective compounds or that the method is not sufficiently sensitive due to different reaction rates.

An alternative approach of decomposition for AcEP includes the process of cycloreversion [40] under reformation of the parent Ac by releasing singlet oxygen (Figure 6), which could be also biologically active. While for DPAcEP, pPyBuAcEP, and mPyBuAcEP, cycloreversion was observed at 70 °C in EtOH, oPyAcEP did not decompose to its parent compound indicating more complex thermal reaction pathways (Figure 7). This suggests for DPAcEP, pPyBuAcEP, and mPyBuAcEP, the formation of carbon-centered radicals occurs in parallel with a slow decomposition by cycloreversion, which could trigger biological actions by the release of singlet oxygen.

As the methods used in chemical systems do not have sufficient sensitivity for the application in *Leishmania*, we explored the role of the radicals and iron in LtP indirectly by studying the influence of a thiol antioxidant and an iron chelator on the IC_50_ values of the EP in LtP viability assays (Figure 8). Interestingly, most of the tested EP exhibited higher IC_50_ values in the presence of the thiol NAC than in its absence (Figure 8A). This clearly indicates that in these compounds (1062MeAcEP, mPyBuAcEP, oPyAcEP, and oPy2C8AcEP) the radical formation is essential for the pharmacological activity and can be prevented by radical scavenging thiols. Why DPAcEP and oOMeAcEP did not respond in a similar way remained unclear. However, in conjunction with heme, thiols could act as reductants for heme iron and, therefore, could theoretically increasingly trigger peroxide cleavage in these compounds. Thus, specific interactions of these compounds may prevent the interference of NAC in these cases. 

The presence of the lipophilic iron chelator PIH in *Leishmania* viability assays (Figure 8B) did show an increase of IC_50_ values for 1062MeAcEP, mPyBuAcEP, DPAcEP, and oOMeAcEP, while for oPyAcEP and oPy2C8AcEP, no increase was observed. As PIH can only chelate iron from the labile iron pool and not from heme compounds, there are still options for alternative activation pathways, for example, via heme.

The formation of radicals derived from EP in *Leishmania* may trigger subsequent events in cellular stress. Since *Leishmania* are lacking catalase and a selenium-dependent glutathione peroxidase system [41,42,43] and have a rather large labile iron pool [16], they appeared to be specifically sensitive with respect to oxidative stress. Furthermore, *Leishmania* are unique in their cellular structure since they possess only one mitochondrion, which is involved in cellular energy production and other metabolic processes. In context to radical formation, mitochondria can be a target but also the trigger for the production of reactive oxygen species. In a first step, we studied the influence of AcEP on the mitochondrial oxygen consumption, which is a good indicator for an intact oxidative phosphorylation system (Figure 9). The results show that most compounds did not strongly influence the oxygen consumption in LtP and J774 macrophages. Thus, inhibition of the mitochondrial electron transfer chain is obviously not a specific property of the peroxyl group per se. However, some compounds, such as mPyBuAcEP and pPyBuAcEP and their analogs strongly inhibited mitochondrial oxygen consumption in LtP (Figure 9A). Interestingly, the non-endoperoxidic analog of mPyBuAcEP (mPyBuAc) exhibited an even stronger inhibition of mitochondrial respiration in LtP, indicating that not the EP group but rather the residual molecular structure contributes to this effect. In context with the results from the other compounds, this suggests that these effects are due to the molecular structure beyond the EP group. Interestingly, the inhibiting effect of these compounds in J774 macrophages is much less pronounced. Possible reasons could be a slightly different target protein but also the fact that macrophages exhibit multiple mitochondria. In a previous study, we have shown that J774 cells, but not LtP, have a significant respiratory reserve capacity [44], which could be used to compensate such events of mitochondrial inhibition.

Since these measurements were carried out after short incubation times with the respective compounds, the effects seen are due to direct inhibition of mitochondria by the compounds, which may subsequently trigger apoptosis [45], but not due to an extrinsic pathway of apoptosis, which secondarily triggers mitochondrial dysfunction and may take several hours to proceed.

Mitochondrial dysfunction (either by direct inhibition or apoptosis-triggered) can lead to the increased formation of superoxide radicals in the respiratory chain [46]. Our tests in LtP were performed by the CMH/EPR method (Figure 10 and Figure 11). To verify the detection system, we performed several control experiments. Buffer samples produce much less CMH oxidation (superoxide production) than LtP. Antimycin A, an inhibitor of mitochondrial complex III [45], significantly boosts superoxide production in *Leishmania*. With respect to AcEP and analogs, only few compounds trigger additional superoxide formation. Interestingly, mPyBuAcEP and its analog increase the superoxide formation by more than 70%. This suggests that this increased superoxide production is linked to the inhibition of mitochondrial oxygen consumption by these compounds as seen in the previous experiment (Figure 9).

## 4. Materials and Methods

### 4.1. Compounds and Reagents

Ac and DPAc were purchased from Aldrich/Merck (Darmstadt, Germany) with a purity > 99%. The following compound’s synthesis and structure characterization were described: AcEP in [47]; DPAcEP in [48]; mPyAc, mPyAcEP, oPyAc, oPyAcEP, pPyBuAc, and pPyBuAcEP in [49]; oOMeAc and oOMeAcEP in [50]; MB359EP in [51]; and 1062MeAcEP in [52]. Compounds BisGluAc, BisGluAcEP, MonoGluAc, MonoGluAcEP, mPyBuAc, mPyBuAcEP, oPy2C8Ac, and oPy2C8AcEP were synthesized and characterized in analogy to procedures published in [49] and identified by ^1^H- and ^13^C-NMR (see Supplement Figures S2–S17). The purity of all synthesized compounds was at least 99%. IUPAC names of Ac/AcEP were listed in Table S1 (Supplement).

Brain heart infusion medium, 5,5-dimethyl-1-pyrroline N-oxide (DMPO), hemin, pentamidine (Pen), antimycin A and resazurin were purchased from Sigma-Aldrich (St. Louis, MO, USA). Diethylenetriaminepentaacetic acid (DTPA), K_2_HPO_4_, KH_2_PO_4_, Na_2_HPO_4_, NaCl, xylenol orange (XO), FeSO_4_, glucose, KCl sodium dithionite, ethanol (EtOH), acetonitrile, and methanol (MeOH) were from Merck (Darmstadt, Germany). Dimethyl sulfoxide (DMSO) and penicillin-streptomycin solution were supplied by VWR (Radnor, PA, USA). Butylated hydroxytoluene was from Roche (Basel, Switzerland). Hydroxyl-3-methoxycarbonyl-2,2,5,5-tetramethylpyrrolidine hydrochloride (CMH) was obtained from Noxygen (Elzach, Germany). Deferoxamine (DFO) was purchased from Novartis (Basel, Switzerland). Dulbecco’s Modified Eagle’s Medium (DMEM) and N-acetyl cysteine (NAC) were produced by Thermo Fisher Scientific (Waltham, MA, USA). Fetal calf serum (FCS) was purchased at Bio & Sell (Nuremberg, Germany). Pyridoxal isonicotinoyl hydrazine (PIH) was produced by Abcam (Cambridge, UK). Yeast extract (YE) was obtained from Amresco (Solon, OH, USA).

### 4.2. Cell Culture

#### 4.2.1. Leishmania Tarentolae Promastigotes (LtP) Cell Culture

LtP (strain P10 from Jena Bioscience, Jena, Germany) were cultivated according to manufacturer instructions up to passage 50 in brain heart infusion medium (37 g/L, pH 7.4) and supplemented with 5 mg/L hemin and 25,000 IU/L penicillin and 25 mg/L streptomycin in 50 mL TubeSpin bioreactors (TPP, Trasadingen. Switzerland). LtP were grown at 27 °C in an incubator (Ehret, Emmendingen, Germany) and agitated at a frequency of 0.05 s^−1^. The parasites were passaged three times a week so that the new culture had a cell density of 18 × 10^6^ LtP/mL or 36 × 10^6^ LtP/mL after each passage. For experiments, LtP were used one day after passage. The cell number was calculated by measuring the optical density (OD) of the culture at 600 nm with a U-1100 photometer (Hitachi Ltd., Tokyo, Japan) and applying the formula × 10^6^ cells/mL = OD_600nm_ × 0.969 × 124 × dilution factor [53].

#### 4.2.2. J774 Macrophage Cell Culture

The murine macrophage cell line J774A.1 (ATCC, TIB-67™, Wesel, Germany) was cultivated up to passage 30 in DMEM (high glucose, 3.7 g/L NaHCO_3_, pH 7.4) with 25,000 IU/L penicillin, 25 mg/L streptomycin, and 10% FCS (heat-inactivated) in 50 mL TubeSpin bioreactors. J774 cells were incubated at 37 °C and 5% CO_2_ on a roller culture apparatus (5 rpm) in a Heraeus Cytoperm 8080 incubator (Thermo Electron Corp., Vienna, Austria). To determine the cell count, the cell suspension was re-suspended and diluted 1:2 with phosphate-buffered saline (PBS): 137 mM NaCl, 1.4 mM KH_2_PO_4_, 4.3 mM Na_2_HPO_4_, and 2.7 mM KCl, pH 7.4. After fixation of the cells by mixing the cell suspension 1:1 with a formaldehyde solution (2%, *v*/*v*), aliquots of the resulting suspension were transferred into a standard Neubauer chamber (VWR, Austria) and counted manually using a light microscope (Motic BA310, Vienna, Austria) at a magnification of 40 times. J774 cells were passaged twice a week in a ratio of 1:10.

### 4.3. Viability Assays

#### 4.3.1. Viability Assay for LtP

Assay medium of YEM (20.7 g/L yeast extract powder, 1.2 g/L K_2_HPO_4_, 0.2 g/L KH_2_PO_4_, 2.9 g/L glucose, pH 7.4) and PBS (1:1, *v*/*v*), 25,000 U/L penicillin, 25 mg/L streptomycin, and 7.7 µM hemin was prepared. Aliquots of 200 µL of this medium and 2 × 10^6^ LtP/mL (where required) were distributed in non-treated cell culture 96-well plates. Compounds (dissolved in DMSO) were added with starting concentrations of 25 µM (Pen) or 200 µM (AcEP/Ac), and then, serial dilutions were carried out resulting in a set of concentrations (25, 5, 1, 0.2, 0.04, and 0.008 µM) or (200, 40, 8, 1.6, 0.32, and 0.064 µM). For each compound concentration, at least triplicates on each plate were performed. After incubation of the plates at 27 °C for 48 h, 50 µL of resazurin solution (final concentration in wells 20 µM) was added to each well and after 4 h of incubation, the fluorescence was measured at 560 nm excitation and 590 nm emission using a plate reader (Perkin Elmer EnSpire, Rodgau, Germany) (adapted from [54,55]). Combination assays for viability in the presence of PIH and NAC were performed the same way. However, one half of the plate was filled with YEM/PBS with 2 × 10^6^ LtP/mL, and the other half of the plate was filled with YEM/PBS with 2 × 10^6^ LtP/mL supplemented with non-toxic concentrations of PIH (50 µM) or NAC (2 mM), so that for each plate, two compounds were tested with and without the presence of PIH or NAC. All tests were repeated at least on four different weeks of cultivation.

#### 4.3.2. Viability Assay for J774 Macrophages

DMEM containing 25,000 U/L penicillin, 25 mg/L streptomycin, 10% FCS, and 1 × 10^5^ J774/mL was prepared. This mixture (200 µL) was distributed in cell culture-treated 96-well plates, except the control wells which contained only DMEM. The cells were incubated for 24 h at 37 °C and 5% CO_2_ to allow attachment. Afterwards the medium and non-adherent cells were removed by washing with PBS, and then, 200 µL DMEM was added to attached cells. Compounds dissolved in DMSO were added in starting concentrations of 25 µM (Pen) or 200 µM (AcEP/Ac), and then, serial dilutions were carried out resulting in the same concentration steps as listed for LtP. For each compound concentration triplicates on each plate were performed. After incubating the plates for 24 h at 37 °C and 5% CO_2_, 50 µL of resazurin solution (final concentration 20 µM) was added, and after 4 h of incubation, the fluorescence was measured at 560 nm excitation and 590 nm emission using a plate reader (Perkin Elmer EnSpire, Rodgau, Germany). All tests were repeated at least on four different weeks of cultivation.

### 4.4. Formation of the Xylenol Orange/Fe^3+^ Complex by EP

XO (125 µM) and butylated hydroxytoluene (4 mM) were dissolved in MeOH/H_2_O (9:1). FeSO_4_ (25 mM) in 2.5 M H_2_SO_4_ was prepared immediately before use (final concentration of 250 µM FeSO_4_ in the color reagent) [56]. An aliquot of 100 µL EP solution (final concentration of 100 µM) was transferred into a disposable cuvette (BRAND, Wertheim, Germany), and 1 mL of the color reagent was added. The EP that reacted slower were incubated for 0, 30, 60, and 90 min at 25 °C. The EP that reacted faster were measured every 5 s over 5 min. At these time points, the OD difference of the sample at 560 nm and 650 nm was determined using a MS1501 UV–VIS diode array spectrophotometer (Shimadzu, Kyoto, Japan). As reference, a mixture of MeOH/H_2_O (9:1) was used [56]. The slope of the reaction rate was calculated using an extinction coefficient of 1.5 × 10^4^ M^−1^ cm^−1^.

### 4.5. UV Detection of Cycloreversion in AcEP

A sample of the corresponding Ac in 1.5 mL EtOH (final concentrations 2, 8, or 10 µM) was transferred into a quartz cuvette and measured by an UV–VIS spectrometer (U3300, Hitachi, Tokyo, Japan) at 70 °C. Afterwards, an aliquot of the corresponding AcEP dissolved in EtOH (final concentration 40 µM) was pipetted into a cuvette, and its UV spectra were measured at 70 °C in a time interval of 1 h over a period of 23 h. After measurements, the re-appearance of the Ac absorption pattern was verified.

### 4.6. Electron Paramagnetic Resonance (EPR) Spectroscopy

#### 4.6.1. Spin Trapping

For EPR spin trapping, 100 µL purified water with 40 mM DMPO, 500 µM AcEP (dissolved in DMSO), and 500 µM FeSO_4_ was prepared [16]. Samples containing DMSO instead of AcEP were used as negative controls. After an incubation period of 5 min at 25 °C, 50 µL of the mixture were aspirated into a disposable glass micropipette (BLAUBRAND intraMARK, BRAND, Wertheim, Germany) and inserted into the split ring resonator (Bruker MD5) of the EPR spectrometer (Bruker Digital Upgrade EMX, Rheinstetten, Germany). For EPR spin trapping at 50 °C, 100 µL H_2_O/acetonitrile (8:2) with 40 mM DMPO, 4 mM AcEP and 1 mM FeSO_4_ were prepared. After an incubation period of 10 min, the whole mixture was aspirated into two 50 µL glass micropipettes and inserted into the split ring resonator of the EPR spectrometer. Then the measurements were started with the following parameters: microwave frequency 9.685 GHz; microwave power 20 mW; modulation frequency 100 kHz; modulation amplitude 2 G; time constant 0.164 s; center field 3448 G; sweep width 100 G; scan time 84 s; attenuation 70 dB; scans 3.

The obtained spectra of the EPR spin trapping experiments were simulated in the software WINSIM 0.98 [57] until a sufficient fit was achieved, and derived coupling parameters were used for assignment of the spin adducts.

#### 4.6.2. Detection of Superoxide Radicals by CMH

An aliquot of the LtP cell culture equivalent to 5 × 10^8^ LtP was transferred into a 1.5 mL Eppendorf tube and was centrifuged (Hettich Universal 16, 20 °C, 2000× *g*, 10 min). After removal of the supernatant, the cell pellet was re-suspended in 1 mL PBS and centrifuged again and the supernatant discarded. The cell pellet was re-suspended in 1 mL of PBS with 15 mM glucose (PBS-gluc), giving a final cell density of 5 × 10^8^ LtP/mL. AcEP/Ac compounds were added (100 µM final concentration) to aliquots of 50 µL of this cell suspension in 1.5 mL Eppendorf tubes and incubated for 20 min at 25 °C. Then, DFO (100 μM final concentration), DTPA (25 μM final concentration), and detection agent CMH (400 µM final concentration) were added to the aliquots before measurement [58]. Samples containing only buffer (PBS-gluc) or only LtP (in PBS-gluc) were used as negative controls, while samples containing 20 μM AA were used as positive control. An aliquot of 17 µL suspension was aspirated into a PTFE tube (ø 0.7 mm). The tube was folded into a quartz tube (ø 4 mm outer diameter) and then transferred to the MD5 resonator of the EPR spectrometer. The measurement was started using following parameters: microwave frequency 9.682 GHz; microwave power 20 mW, modulation frequency 100 kHz; modulation amplitude 1 G; time constant 0.82 s; center field 3448 G; sweep width 100 G; scan time 84 s; attenuation 70 dB; separate scans 5. Based on a calibration curve with carboxy proxyl (CP^●^) changes of the peak-to-peak intensity of CM^●^ spectra were converted to CMH oxidation rates (superoxide radical generation).

### 4.7. O_2_ consumption of Cells

Oxygen consumption rates of LtP in complete BHI culture medium and J774 in PBS-gluc treated with AcEPs were measured using round-bottomed OxoPlates (OP96U, Precision Sensing GmbH, Regensburg, Germany). An aliquot of corresponding medium (BHI or PBS-gluc) was transferred to an open Erlenmeyer flask and was stirred on a magnetic stirrer for 30 min at 25 °C to saturate it with air. Another aliquot was mixed with approximately 50 mg of the reducing agent sodium dithionite in a tightly closed falcon tube to remove the dissolved oxygen. Aliquots of 200 µL of both calibration media (air-saturated or oxygen-free) were distributed to 6 wells each of the first row of an OxoPlate. To prevent oxygen exchange with the environment, 70 µL of paraffin oil were layered on top of each well containing liquid. Then, the fluorescence of both calibration media was measured at a microplate reader (Perkin Elmer EnSpire, Rodgau, Germany) in a dual kinetic mode, using an excitation wavelength of 540 nm for both dyes and emission wavelengths of 650 nm for the indicator dye and 590 nm for the reference dye. The ratio of both fluorescence intensities (I_R_) was calculated for each well and averaged giving the two calibration constants k_0_ (oxygen-free) or k_100_ (air-saturated). From the second row on each well was filled with 50 µL air-saturated medium. The respective amounts of AcEP/Ac were added and mixed properly. LtP (140 × 10^6^ cells/mL) or J774 (2 × 10^6^ cells/mL) were suspended in air-saturated BHI or PBS-glu, respectively. Aliquots of 150 µL were transferred to each well giving a final concentration of the AcEP/Ac of 200 µM. On top of each well, 70 µL of paraffin oil was added. Both fluorescence intensities of each well were determined in 5 min intervals over up to 40 min. Each compound was measured in quadruplicates. The oxygen concentration was calculated using the following equation according to the manual of the manufacturer (Precision Sensing GmbH):

[O_2_] (µM) = 100 × (k_0_/I_R_ − 1)/(k_0_/k_100_ − 1) × 2.83

The slope of the change of the oxygen concentration over time was calculated giving the oxygen consumption rate [µM/min] for each well. The value obtained for the respective air-saturated media was subtracted. The rate was finally normalized to the oxygen consumption rate of the wells that did contain cells but no test compounds.

### 4.8. Structures

All 2D structures were drawn in BIOVIA Draw 2021 (Dassault Systems, Vélizy-Villacoublay, France). For 3D modeling of mPyBuAcEP and mPyBuAc, mol files were exported to Hyperchem 7.5 (Hypercube). There basic 3D structures were built, and then, structure optimization using the semiempirical PM3 method was performed.

### 4.9. Data Analysis

Calculations were carried out using Microsoft Excel 2016 (Microsoft). The graphs were created using Origin 6.1 (OriginLab Corporation, Northampton, MA, USA). For all groups of experimental data, mean and standard deviation (SD) were calculated. Statistically significant differences between groups of sample data were identified by unpaired two-tailed Student’s *t*-test. Differences on the level of *p* < 0.05 were considered significant. The number of replicates is indicated at the respective figures/tables. The half-maximal inhibitory concentrations (IC_50_) were determined from non-linear concentration-response curves using a four-parameter logistic model [59] and expressed as the mean ± SD.

## 5. Conclusions

According to structure activity correlations (Figure 12), effective antileishmanial compounds (IC_50_ in viability assays less than 10 µM) have a predicted LogP_OW_ higher than 3.5 and a PSA smaller than 100 Å^2^. Furthermore, a rapid reaction rate with iron (II) in chemical systems is not correlated with a low IC_50_ value in viability assays for *Leishmania*. However, the faster the reaction of EP with iron (II) was, the more important is the EP group obviously for its pharmacological action. This does not exclude a contribution of the EP group to the antileishmanial activity in slowly reacting EP, as was shown by the interference of the iron chelator PIH.

Based on the antileishmanial effect and the calculated selectivity with respect to macrophages, the compounds mPyBuAcEP (IC_50,LtP_ = 0.70 µM, IC_50,J774_ = 24.6 µM) and mPyBuAc (IC_50,LtP_ = 0.54 µM, IC_50,J774_ = 74.2 µM) appear to be the most favorable substances for future research. In case these substances would confirm their activity in intracellular *Leishmania donovani*, they would meet the hit selection criteria of IC_50_ values < 10 µM as defined by Katsuno et al. [60] for the development of drugs against visceral leishmaniasis.

The endoperoxide mPyBuAcEP reacts with iron, while no DMPO spin adduct could be detected under our conditions. In parallel, this compound was shown to undergo cycloreversion, which could contribute to its antileishmanial action by the release of singlet oxygen. Mechanistic data in LtP show that mPyBuAcEP actions on *Leishmania* can be prevented by the radical scavenger NAC and the iron chelator PIH, suggesting the involvement of radical formation in its pharmacological activity. However, the observation that the non-endoperoxidic analog mPyBuAc is as efficient as the EP against *Leishmania* suggests that the molecular structure beyond the EP group is important for its pharmacological action. In this context, one should realize that not only the presence/absence of the EP group makes the structural difference in biological activity between those molecules, but the introduction of the peroxyl group distorts the whole anthracene ring system. This conformational change on top of the presence of the EP group may also play a role in the slightly different activity.

Therefore, future experiments should reveal the actual targets of this mPyBuAcEP/mPyBuAc pair as well as potential host cell toxicity. Subsequently, tests in more complex model systems for pathogenic *Leishmania* and in vivo models are required.

## Figures and Tables

**Table 1:** Bioavailability parameters of compounds generated with the software BIOVIA Draw (MW = molecular weight, LogP_OW_ = partition coefficient, HAccept = hydrogen bond acceptors, stereo centers, PSA = polar surface area).

Compound	Sum Formula	MW (g/mol)	LogP_OW_	HAccept	Stereo Centers	PSA (Å^2^)
Ac	C_14_H_10_	178.2	3.64	0	0	0
AcEP	C_14_H_10_O_2_	210.2	2.62	2	0	18.4
1062MeAcEP	C_26_H_22_N_2_O_2_ × 2CF_3_O_3_S	692.6	3.67	8	0	157.3
BisGluAc	C_42_H_46_O_20_	870.8	2.33	20	10	247.3
BisGluAcEP	C_42_H_46_O_22_	902.8	1.38	22	10	265.7
DPAc	C_26_H_18_	330.4	6.68	0	0	0
DPAcEP	C_26_H_18_O_2_	362.4	5.50	2	0	18.4
MB359EP	C_42_H_52_O_20_	876.8	1.03	20	12	247.3
MonoGluAc	C_28_H_28_O_10_	524.5	2.99	10	5	123.6
MonoGluAcEP	C_28_H_28_O_12_	556.5	2.00	12	5	142.1
mPyAc	C_24_H_16_N_2_	332.3	4.38	2	0	25.7
mPyAcEP	C_24_H_16_N_2_O_2_	364.3	3.20	4	0	44.2
mPyBuAc	C_28_H_24_N_2_	388.5	5.78	2	0	25.7
mPyBuAcEP	C_28_H_24_N_2_O_2_	420.5	4.60	4	2	44.2
oOMeAc	C_28_H_22_O_2_	390.4	6.65	2	0	18.4
oOMeAcEP	C_28_H_22_O_4_	422.4	5.47	4	0	36.9
oPyAc	C_24_H_16_N_2_	332.3	5.23	2	0	25.7
oPyAcEP	C_24_H_16_N_2_O_2_	364.3	4.06	4	0	44.2
oPy2C8Ac	C_34_H_34_N_2_O_2_	502.6	8.41	4	0	52.0
oPy2C8AcEP	C_34_H_34_N_2_O_4_	534.6	7.24	6	2	70.5
pPyBuAc	C_28_H_24_N_2_	388.5	5.78	2	0	25.7
pPyBuAcEP	C_28_H_24_N_2_O_2_	420.5	3.98	4	4	44.2

**Table 2:** IC_50_ values of AcEP and their corresponding non-EP analogues (Ac). The IC_50_ values were determined using resazurin viability assays of serial dilutions in 96-well plates of the respective compounds with an endpoint of 48 h and starting with 2 × 10^6^ LtP/mL or 10^5^ J774 cells/mL. Compounds were tested in the concentration range from 0.008 µM to 200 µM. Data represent mean ± SD of 4–8 individual experiments.

Compound	LtP IC_50_ (µM)	J774 IC_50_ (µM)	Selectivity IC_50,J774_/IC_50,LtP_
Pen	0.52 ± 0.16	12.27 ± 3.96	23.6
Ac	>200	>200	n.d.
AcEP	19.7 ± 14.1	1.21 ± 0.32	0.06
1062MeAcEP	62.0 ± 34.2	>200	>3.2
BisGluAc	>200	>200	n.d.
BisGluAcEP	>200	>200	n.d.
DPAc	197 ± 92	136 ± 35	0.7
DPAcEP	9.67 ± 2.15	197 ± 35	20
MB359EP	>200	>200	n.d.
MonoGluAc	158.3 ± 45.1	101.4 ± 7.8	0.6
MonoGluAcEP	32.3 ± 23.4	152.6 ± 82.1	4.7
mPyAc	52.5 ± 3.3	63.7 ± 7.6	1.2
mPyAcEP	>200	>200	n.d.
mPyBuAc	0.54 ± 0.09	74.2 ± 45.9	139
mPyBuAcEP	0.70 ± 0.31	24.6 ± 7.3	35
oOMeAc	73.3 ± 25.2	>200	>2.7
oOMeAcEP	40.7 ± 12.3	>200	>4.9
oPyAc	34.5 ± 12.8	70.0 ± 10.5	2.0
oPyAcEP	1.84 ± 0.13	2.24 ± 0.08	1.2
oPy2C8Ac	11.3 ± 3.4	34.8 ± 10.5	3.0
oPy2C8AcEP	0.81 ± 0.37	1.28 ± 0.46	1.6
pPyBuAc	1.86 ± 1.10	107 ± 46	57
pPyBuAcEP	0.707 ± 0.662	26.1 ± 10.6	37

**Table 3:** Formation of the xylenol/Fe^3+^ complex by AcEP and 250 µM FeSO_4_ in methanol/H_2_O (9:1). Data represent mean ± SD of 3 experiments.

Compound	XO/Fe^3+^ Formation Rate (nmol/min)	Log10 (Formation Rate)	IC_50,non-EP_/IC_50,EP_
AcEP	8110 ± 4197	3.90	>10.1
1062MeAcEP	609 ± 27	2.78	-
BisGluAcEP	6800 ± 326	3.83	1
DPAcEP	677 ± 86	2.83	20.3
MB359EP	18,533 ± 1147	4.26	-
MonoGluAcEP	12,697 ± 1500	4.10	4.89
mPyAcEP	708 ± 37	2.85	<0.26
mPyBuAcEP	731 ± 111	2.86	0.77
oOMeAcEP	545 ± 17	2.73	1.79
oPyAcEP	81,700 ± 11,921	4.91	18.7
oPy2C8AcEP	42,667 ± 6302	4.63	14.0
pPyBuAcEP	67 ± 3	1.82	2.63

**Table 4:** Coupling constants obtained from simulation of DMPO spin adduct EPR spectra in the presence of AcEP by using the program WINSIM. a_H_ = proton hyperfine coupling constant; a_N_ = nitrogen hyperfine coupling constant. (Red., reductant; Int., relative intensity).

Compound	Red.	Most Abundant Species				
		Int. (%)	a_N_ (G)	a_H_ (G)	a_N_/a_H_	Assignment
MonoGluAcEP	Fe^2+^	75	15.8	23.9	0.66	DMPO/^●^C
		25	15.0	14.5	1.03	DMPO/^●^OH
oPy2C8AcEP	Fe^2+^	100	15.7	23.3	0.67	DMPO/^●^C
oPyAcEP	Fe^2+^	100	15.7	23.3	0.67	DMPO/^●^C
MB359EP	Fe^2+^	100	16.1	22.8	0.70	DMPO/^●^C

## Data Availability

Data collected during the present study are available from the corresponding author.

## Supplementary Materials

The following supporting information can be downloaded at: https://www.mdpi.com/article/10.3390/molecules27206846/s1. Table S1: Compound abbreviations and IUPAC names. Figure S1: UV spectra of Ac compounds. Figure S2–S17: ^1^H- and ^13^C-NMR spectra of BisGluAc, BisGluAcEP, MonoGluAc, MonoGluAcEP, mPyBuAc, mPyBuAcEP, oPy2C8Ac, and oPy2C8AcEP, respectively.

## Sample Availability

Samples of the compounds are available from the authors.
