# Supplementary material for: Antileishmanial Anthracene Endoperoxides: Efficacy In Vitro, Mechanisms and Structure-Activity Relationships
Source: Molecules. 2022 Oct 13;27(20):6846. doi: 10.3390/molecules27206846 (PMC9612231; doi:10.3390/molecules27206846)
Supplement: Supplementary file 1 [file molecules-27-06846-s001.zip › molecules-1934949-supplementary.pdf]

Supporting Information for

Antileishmanial Anthracene Endoperoxides: Efficacy in vitro, Mechanisms and Structure-Activity Relationships

Laura Machin[1,2], Martin Piontek[1] , Sara Todhe[1] , Katrin Staniek[1] , Lianet Monzote[3] , Werner Fudickar[4] , Torsten Linker[4] and Lars Gille[1,*]

1 Institute of Pharmacology and Toxicology, Department of Biomedical Sciences, University of Veterinary Medicine, 1210 Vienna, Austria

2 Pharmacy Department, Institute of Pharmacy and Food Sciences, University of Havana, 13600 Havana, Cuba

3 Parasitology Department, Institute of Tropical Medicine "Pedro Kouri", 11400 Havana, Cuba

4 Department of Organic Chemistry, Institute of Chemistry, University of Potsdam, 14476 Golm/Potsdam, Germany

*Correspondence: Lars.Gille@vetmeduni.ac.at; Tel.: +43125077-2907

Table of contents

Fig./Tab.	Caption	Page
	NMR assignments	3
Tab. S1	List of abbreviations and IUPAC names of all AcEPs and analogues (Ac).	5
Fig. S1	UV spectra of non-endoperoxidic Ac analogs (40 µM). (A) DPAc, (B) oPyAc, (C) pPyBuAc and (D) mPyBuAc.	6
Fig. S2	The ^{1}H NMR spectrum of BisGluAc	7
Fig. S3	The ^{13}C NMR spectrum of BisGluAc	8
Fig. S4	The ^{1}H NMR spectrum of BisGluAcEP	9
Fig. S5	The ^{13}C NMR spectrum of BisGluAcEP	10
Fig. S6	The ^{1}H NMR spectrum of MonoGluAc	11
Fig. S7	The ^{13}C NMR spectrum of MonoGluAc	12
Fig. S8	The ^{1}H NMR spectrum of MonoGluAcEP	13
Fig. S9	The ^{13}C NMR spectrum of MonoGluAcEP	14
Fig. S10	The ^{1}H NMR spectrum of mPyBuAc	15
Fig. S11	The ^{13}C NMR spectrum of mPyBuAc	16
Fig. S12	The ^{1}H NMR spectrum of mPyBuAcEP	17
Fig. S13	The ^{13}C NMR spectrum of mPyBuAcEP	18
Fig. S14	The ^{1}H NMR spectrum of oPy2C8Ac	19
Fig. S15	The ^{13}C NMR spectrum of oPy2C8Ac	20
Fig. S16	The ^{1}H NMR spectrum of oPy2C8AcEP	21
Fig. S17	The ^{13}C NMR spectrum of oPy2C8AcEP	22

NMR Spectroscopic Data

General. ^{1}H NMR and ^{13}C NMR spectra were measured using a Bruker NEO 400, Bruker MAS 400, or Bruker NEO 500 (Rheinstetten, Germany) NMR spectrometer. Chemical shifts and multiplicities were derived from corresponding ^{13}C APT or HSQC measurements.

9,10-Bis-(1-O-2,3,4,6-tetra-O-acetyl-β-D-glucopyranosyl)-anthracene (BisGluAc). ^{1}H NMR (500 MHz, $CDCl_3$): δ = 1.80 (2s, 6H; 6-OAc), 2.01 (2s, 6H; 4-OAc), 2.08 (2s, 6H; 3-OAc), 2.23 (2s, 6H; 2-OAc), 3.52 (2ddd, *J* = 9.9, 5.5, 2.7 Hz, 2H; 5-H), 3.89 (2dd, *J* = 12.1, 2.7 Hz, 2H; 6a-H), 4.23 (2dd, *J* = 12.1, 5.6 Hz, 2H;6b-H), 5.21 (2d, *J* = 8.1 Hz, 2H; 1-H), 5.24 (2dd, *J* = 9.9, 9.4 Hz, 2H; 4-H), 5.33 (2dd, *J* = 9.7, 9.4 Hz, 2H; 3-H), 5.64 (2dd, *J* = 9.7, 8.1 Hz, 2H; 2-H), 7.47–7.50 (m, 4H; 2-ANTH, 3-ANTH, 6-ANTH, 7-ANTH), 8.38–8.41 ppm (m, 4H; 1-ANTH, 4-ANTH, 5-ANTH, 8-ANTH). ^{13}C NMR (125 MHz, $CDCl_3$): δ = 20.5 (2q; OAc-6), 20.6 (2q; OAc-4), 20.7 (2q; OAc-3), 20.9 (2q; OAc-2), 61.8 (2t; C-6), 68.7 (2d; C-4), 71.9 (2d; C-5), 72.2 (2d; C-2), 73.1 (2d; C-3), 102.8 (2d; C-1), 122.7 (4d; ANTC-1, ANTC-4, ANTC-5, ANTC-8), 125.3 (4s; ANTC-11, ANTC-12, ANTC-13, ANTC-14), 125.7 (4d; ANTC-2, ANTC-3, ANTC-6, ANTC-7), 144.9 (2s; ANTC-9, ANTC-10), 169.4 (2s; OAc-2), 169.5 (2s; OAc-4), 170.4 (2s; OAc-6), 170.5 ppm (2s; OAc-3).

9,10-Bis-(1-O-2,3,4,6-tetra-O-acetyl-β-D-glucopyranosyl)-9,10-dihydro-9,10-epidioxidoanthracene (BisGluACEP). ^{1}H NMR (500 MHz, $CDCl_3$): δ = 2.03 (s, 6H; 2-OAc), 2.05 (s, 6H; 3-OAc), 2.06 (s, 6H; 4-OAc), 2.13 (s, 6H; 6-OAc), 3.86 (ddd, *J* = 10.0, 5.7, 2.3 Hz, 2H; 5-H), 4.25 (dd, *J* = 12.3, 2.2 Hz, 2H; 6a-H), 4.31 (dd, *J* = 12.3, 5.7 Hz, 2H; 6b-H), 5.21 (dd, *J* = 10.0, 9.2 Hz, 2H; 4-H), 5.34 (dd, *J* = 9.6, 9.2 Hz, 2H; 3-H), 5.38 (dd, *J* = 9.6, 7.6 Hz, 2H; 2-H), 5.47 (d, *J* = 7.6 Hz, 2H; 1-H), 7.28 (dd, *J* = 8.5, 7.2 Hz, 2H; 2-ANTH, 7-ANTH), 7.33 (dd, *J* = 8.5, 7.6 Hz, 2H; 3-ANTH, 6-ANTH), 7.59 (d, *J* = 7.6 Hz, 2H; 4-ANTH, 5-ANTH), 7.86 ppm (d, *J* = 7.2 Hz, 2H; 1-ANTH, 8-ANTH). ^{13}C NMR (125 MHz, $CDCl_3$): δ = 20.7 (q; OAc-3), 20.7 (q; OAc-4), 20.9 (q; OAc-2), 20.9 (q; OAc-6), 62.3 (t; C-6), 68.5 (d; C-4), 71.1 (d; C-2), 72.4 (d; C-5), 72.6 (d; C-3), 96.6 (d; C-1), 102.7 (s; ANTC-9, ANTC-10), 120.2 (d; ANTC-4, ANTC-5), 121.3 (d; ANTC-1, ANTC-8), 127.9 (d; ANTC-2, ANTC-7), 128.3 (d; ANTC-3, ANTC-6), 136.7 (s; ANTC-11, ANTC-12), 138.4 (s; ANTC-13, ANTC-14), 169.5 (s; OAc-2), 169.6 (s; OAc-4), 170.2 (s; OAc-3), 170.6 ppm (s; OAc-6).

9-(1-O-2,3,4,6-Tetra-O-acetyl-β-D-glucopyranosyl)-anthracene (MonoGluAc). ^{1}H NMR (500 MHz, $CDCl_3$): δ = 1.77 (s, 3H; 6-OAc), 2.00 (s, 3H; 4-OAc), 2.08 (s, 3H; 3-OAc), 2.23 (s, 3H; 2-OAc), 3.49 (ddd, *J* = 10.0, 5.7, 2.6 Hz, 1H; 5-H), 3.83 (dd, *J* = 12.1, 2.6 Hz, 1H; 6a-H), 4.22 (dd, *J* = 12.1, 5.7 Hz, 1H; 6b-H), 5.23 (d, *J* = 8.1 Hz, 1H; 1-H), 5.24 (dd, *J* = 10.0, 9.3 Hz, 1H; 4-H), 5.32 (dd, *J* = 9.7, 9.3 Hz, 1H; 3-H), 5.65 (dd, *J* = 9.7, 8.1 Hz, 1H; 2-H), 7.44–7.48 (m, 4H; 2-ANTH, 3-ANTH, 6-ANTH, 7-ANTH), 7.95–7.99 (m, 2H; 4-ANTH, 5-ANTH), 8.28 (s, 1H; 10-ANTH), 8.38–8.42 ppm (m, 2H; 1-ANTH, 8-ANTH). ^{13}C NMR (125 MHz, $CDCl_3$): δ = 20.4 (q; OAc-6), 20.7 (q; OAc-4), 20.8 (q; OAc-3), 21.0 (q; OAc-2), 61.9 (t; C-6), 68.8 (d; C-4), 71.9 (d; C-5), 72.3 (d; C-2), 73.2 (d; C-3), 102.7 (d; C-1), 122.8 (2d; ANTC-1, ANTC-8), 124.0 (d; ANTC-10), 125.0 (2s; ANTC-11, ANTC-12), 125.5 (2d; ANTC-2, ANTC-7), 125.6 (2d; ANTC-3, ANTC-6), 128.1 (2d; ANTC-4, ANTC-5), 132.2 (2s; ANTC-13, ANTC-14), 147.5 (s; ANTC-9), 169.4 (s; OAc-2), 169.5 (s; OAc-4), 170.4 (s; OAc-6), 170.5 ppm (s; OAc-3).

9-(1-O-2,3,4,6-Tetra-O-acetyl-β-D-glucopyranosyl)-9,10-dihydro-9,10-epidioxidoanthracene (MonoGluAcEP). ^{1}H NMR (500 MHz, $CDCl_3$): *δ* = 1.97 (s, 3H; 2-OAc), 2.02 (s, 3H; 3-OAc), 2.03 (s, 3H; 4-OAc), 2.11 (s, 3H; 6-OAc), 3.88 (ddd, *J* = 10.1, 6.0, 2.4 Hz, 1H; 5-H), 4.22 (dd, *J* = 12.3, 2.4 Hz, 1H; 6_a-H), 4.30 (dd, *J* = 12.3, 6.0 Hz, 1H; 6_b-H), 5.18 (dd, *J* = 10.1, 9.3 Hz, 1H; 4-H), 5.32 (dd, *J* = 9.7, 9.3 Hz, 1H; 3-H), 5.34 (dd, *J* = 9.7, 7.7 Hz, 1H; 2-H), 5.50 (d, *J* = 7.7 Hz, 1H; 1-H), 5.99 (s, 1H; 10-ANTH), 7.31−7.36 (m, 4H; 2-ANTH, 3-ANTH, 6-ANTH, 7-ANTH), 7.42−7.46 (m, 2H; 4-ANTH, 5-ANTH), 7.58−7.59 (m, 1H; 1-ANTH), 7.89−7.92 ppm (m, 1H; 8-ANTH); ^{13}C NMR (125 MHz, $CDCl_3$): *δ* = 20.8 (q; OAc-3),

20.8 (q; OAc-4), 20.9 (q; OAc-2), 21.0 (q; OAc-6), 62.6 (t; C-6), 68.8 (d; C-4), 71.5 (d; C-2), 72.6 (d; C-3), 72.9 (d; C-5), 80.4 (d; ANTC-10), 96.9 (d; C-1), 103.5 (s; ANTC-9), 120.5 (d; ANTC-1), 121.6 (d; ANTC-8), 123.5 (d; ANTC-5), 123.6 (d; ANTC-4), 128.2 (d; ANTC-7), 128.3 (d; ANTC-3), 128.4 (d; ANTC-2), 128.6 (d; ANTC-6), 137.6 (s; ANTC-11), 138.3 (s; ANTC-12), 138.6 (s; ANTC-13), 139.7 (s; ANTC-14), 169.7 (s; OAc-2), 169.9 (s; OAc-4), 170.2 (s; OAc-3), 170.7 ppm (s; OAc-6).

9,10-Bis-(3-pyridyl)-2-*tert*butylanthracene (mPyBuAc). ^{1}H NMR (400 MHz, $CDCl_3$): δ = 1.29 (s, 9H, *t*Bu), 7.38−7.41 (m, 2H, 6-ANTH, 7-ANTH), 7.41 (d, *J* = 8.9 Hz, 1H, 3-ANTH), 7.51 (s, 1H, 1-ANTH), 7.54−7.61 (m, 5H, 4-ANTH, 5-ANTH, 8-ANTH, 5-HPy, 5'-HPy), 7.60−7.65 (m, 2H, 4-HPy, 4'-HPy), 8.77 (br, m, 2H, 2-HPy), 8.86 ppm (br, m, 2H, 6-HPy, 6'-HPy). ^{13}C NMR (100 MHz, $CDCl_3$): δ = 30.69 (q, *t*Bu), 35.04 (s, *t*Bu), 120.5 (d, ANTC-1), 123.4 (d, PyC-5, PyC-5'), 125.45 (d, ANTC-6), 125.6 (d, ANTC-4), 126.1 (d, ANTC-7), 126.3 (d, ANTC-5), 126.4 (d, ANTC-3), 128.4 (s, ANTC-9), 128.5 (s, ANTC-11), 128.7 (s, ANTC-10), 129.8 (s, ANTC-12), 130.1 (s, ANTC-9), 130.2 (s, ANTC-10), 132.0 (s, ANTC-13), 132.1 (s, ANTC-14), 133.1 (s, PyC-3, PyC-3'), 133.3 (s, ANTC-2), 134.4 (d, PyC-4), 134.8 (d, PyC-4'), 138.8 (d, ANTC-8), 148.2 (d, PyC-6, PyC-6'), 151.6 ppm (d, PyC-2, PyC-2').

9,10-Bis-(3-pyridyl)-2-*tert*butyl-9,10-dihydro-9,10-epidioxidoanthracene (mPyBuAcEP). ^{1}H NMR (400 MHz, $CDCl_3$): δ = 1.20 (s, *t*Bu), 7.08 (d, *J* = 8.2 Hz, 1H, 3-ANTH), 7.13 (m, 2H, 6-ANTH, 7-ANTH), 7.14 (s, 1H, 1-ANTH), 7.26 (m, 2H, H-ANT5, H-ANT8), 7.27 (d, *J* = 8.2 Hz, 1H, 4-ANTH), 7.60−7.63 (m, 2H, 4-HPy, 4'-HPy), 8.05−8.10 (m, 2H, 5-HPy, 5'-HPy), 8.85 (br, m, 2H, 2-HPy, 2'-HPy), 9.01−9.03 ppm (m, 2H, 6-HPy, 6'-HPy). ^{13}C NMR (100 MHz, $CDCl_3$): δ =31.2 (q, *t*Bu), 34.9 (s, *t*Bu), 82.9 (s, ANTC-9), 83.3 (s, ANTC-10), 120.3 (d, ANTC-1, ANTC-8), 123.0 (d, PyC-5, PyC-5', ANTC-3, ANTC-5), 123.2 (d, ANTC-4), 124.9 (d, ANTC-6, ANTC-7), 128.0 (d, PyC-4, PyC-4'), 128.1 (s, ANTC-2),136.4 (s, ANTC-11, ANTC-14), 138.7 (s, ANTC-12, ANTC-13), 139.7 (s, PyC-3, PyC-3'), 148.9 (d, PyC-6, PyC-6'), 151.6 ppm (d, PyC-2, PyC-2').

9,10-Bis-(2-pyridyl)-2-(nonyl-2-oxo)-anthracene (oPy2C8Ac). ^{1}H NMR (400 MHz, $CDCl_3$): δ = 0.87 (t, *J* = 6.3 Hz, 3H, AlkylH-9), 1.29 (m, 8H, 5-AlkylH−8-AlkylH), 1.59 (m, 2H, 4-AlkylH), 2.30 (t, *J* = 7.4 Hz, 2H, 3-AlkylH), 5.14 (s, 2H, 1-AlkylH), 7.32 (s, 1H, 1-ANTH), 7.33−7.40 (m, 2H, 6-ANTH, 7-ANTH), 7.50−7.59 (m, 8H, 3-ANTH, 4-ANTH, 3-HPy, 3'-HPy, 4-HPy, 4'-HPy, 5-HPy, 5'-HPy), 7.99−8.01 (m, 2H, 5-ANTH, 8-ANTH), 8.98−9.01 ppm (6-HPy, 6'-HPy). ^{13}C NMR (100 MHz, $CDCl_3$): δ = 14.0 (q, AlkylC-9), 22.5 (t, AlkylC-8), 24.7 (t, AlkylC-7), 28.9 (t, AlkylC-6), 29.0 (t, AlkylC-5), 31.6 (t, AlkylC-4), 34.0 (t, AlkylC-3), 66.2 (t, AlkylC-1), 122.7 (d, ANTC-1, ANTC-8), 125.1 (d, PyC-4, PyC-4'), 125.5 (d, ANTC-4), 125.8 (d, PyC-3, PyC-3'), 126.2 (d, PyC-5, PyC-5'), 126.8 (d, ANTC-6, ANTC-7), 126.9 (d, ANTC-3, ANTC-5), 127.0 (s, ANTC-2), 127.1 (s, ANTC-11), 129.3 (s, ANTC-12, ANTC-13), 129.9 (s, ANTC-14), 133.2 (s, ANTC-9, ANTC-10), 149.8 (s, PyC-3, PyC-3'), 157.9 (d, PyC-6, PyC-6'), 178.7 ppm (s, AlkylC-2).

9,10-Bis-(2-pyridyl)-2-(nonyl-2-oxo)- 9,10-dihydro-9,10-epidioxidoanthracene (oPy2C8AcEP). ^{1}H NMR (400 MHz, $CDCl_3$): δ = 0.87 (t, *J* = 6.4 Hz, 3H, 9-AlkylH), 1.25 (m, 8H, 5-AlkylH−8-alkylH), 1.56−1.58 (m, 2H, 4-AlkylH), 2.25 (t, *J* = 7.3 Hz, 2H, 3-AlkylH), 5.00 (s, 2H, 1-AlkylH), 7.18−7.21 (m, 7 H, 1-ANTH, 3-ANTH, 4-ANTH, 5-ANTH, 6-ANTH, 7-ANTH, 8-ANTH), 7.48−7.50 (m, 2H, 3-PyH, 3'-PyH), 7.82−7.83 (m, 2H, 4-PyH, 4'-PyH), 7.94−7.95 (m, 2H, 5-PyH, 5'-PyH), 8.88−8.89 ppm (m, 2H, 6-PyH, 6'-PyH). ^{13}C NMR (100 MHz, $CDCl_3$): δ = 14.4 (q, AlkylC-9), 22.9 (t, AlkylC-8), 25.2 (t, AlkylC-7), 29.2 (t, AlkylC-6), 29.4 (t, AlkylC-5), 31.9 (t, AlkylC-4), 34.6 (t, AlkylC-3), 66.0 (t, AlkylC-1), 84.4 (s, ANTC-9, ANTC-10), 122.7 (d, ANTC-5, ANTC-8), 123.4 (d, ANTC-1), 123.6 (d, ANTC-3, ANTC-4), 123.8 (d, ANTC-6, ANTC-7), 127.5 (d, PyC-3, PyC-3'), 127.8 (d, PyC-4, PyC-4'), 127.9 (d, PyC-5, PyC-5'), 135.8 (s, ANTC-11), 137.7 (s, ANTC-14), 137.8 (s, ANTC-12), 140.2 (ANTC-13), 154.4 (d, PyC-6, PyC-6'), 173.8 ppm (s, AlkylC-2).

Table S1: List of abbreviations and IUPAC names of all AcEPs and analogues (Ac).

Abbreviation	IUPAC Name
Ac	anthracene
AcEP	9,10-dihydro-9,10-epidioxidoanthracene
1062MeAcEP	9,10-Bis(1-methylpyridinium-4-yl)-9,10-dihydro-9,10-epidioxidoanthracene trifluoro-methanesulfonate
BisGluAc	9,10-Bis-(1-O-2,3,4,6-tetra-O-acetyl-β-D-glucopyranosyl)-anthracene
BisGluAcEP	9,10-Bis-(1-O-2,3,4,6-tetra-O-acetyl-β-D-glucopyranosyl)-9,10-dihydro-9,10-epidioxidoanthracene
DPAc	9,10-diphenylanthracene
DPAcEP	9,10-diphenyl-9,9-dihydro-9,10-epidioxidoanthracene
MB359EP	2-*tert*-Butyl-1,4-bis-(1-O-2,3,4,6-tetra-O-acetyl-β-D-glucopyranosyl)-1,4-dihydro-1,4-epidioxidonaphthalene
MonoGluAc	9-(1-O-2,3,4,6-Tetra-O-acetyl-β-D-glucopyranosyl)-anthracene
MonoGluAcEP	9-(1-O-2,3,4,6-Tetra-O-acetyl-β-D-glucopyranosyl)-9,10-dihydro-9,10-epidioxidoanthracene
mPyAc	9,10-bis-(3-pyridyl)-anthracene
mPyAcEP	9,10-bis-(3-pyridyl)-9,10-dihydro-9,10-epidioxidoanthracene
mPyBuAc	9,10-Bis-(3-pyridyl)-2-*tert*butylanthracene
mPyBuAcEP	9,10-Bis-(3-pyridyl)-2-*tert*butyl-9,10-dihydro-9,10-epidioxidoanthracene
oOMeAc	9,10-bis(2-methoxyphenyl)-anthracene
oOMeAcEP	9,10-bis(2-methoxyphenyl)-9,10-dihydro-9,10-epidioxidoanthracene
oPyAc	9,10-bis-(2-pyridyl)-anthracene
oPyAcEP	9,10-bis-(2-pyridyl)-9,10-dihydro-9,10-epidioxidoanthracene
oPy2C8Ac	9,10-Bis-(2-pyridyl)-2-(nonyl-2-oxo)-anthracene
oPy2C8AcEP	9,10-Bis-(2-pyridyl)-2-(nonyl-2-oxo)-9,10-dihydro-9,10-epidioxidoanthracene
pPyBuAc	9,10-bis-(4-pyridyl)-anthracene
pPyBuAcEP	9,10-bis-(2-pyridyl)-9,10-dihydro-9,10-epidioxidoanthracene

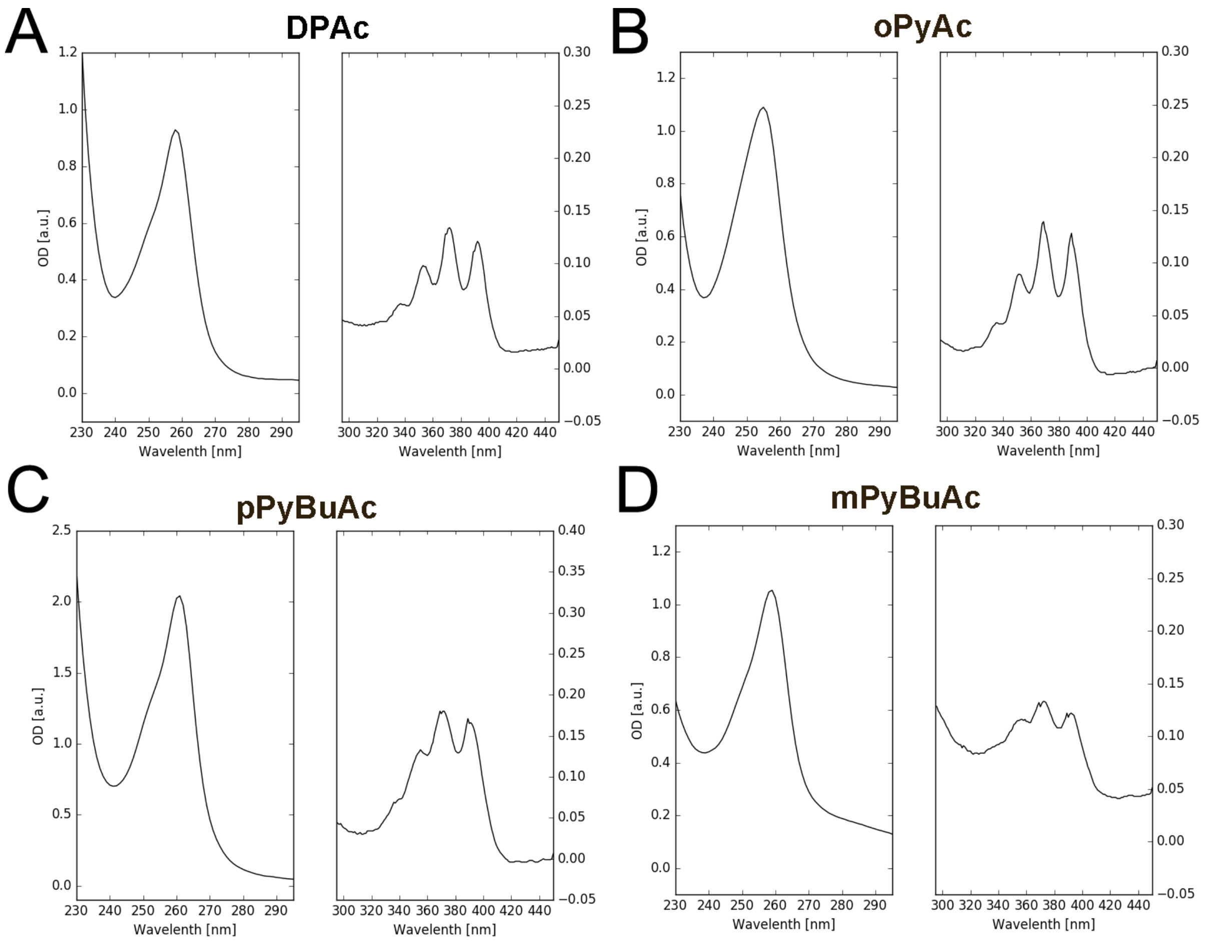

Figure S1: UV spectra of non-endoperoxidic Ac analogs (40 μM). (A) DPAc, (B) oPyAc, (C) pPyBuAc and (D) mPyBuAc.

Figure S2

^1H NMR (500 MHz) spectrum

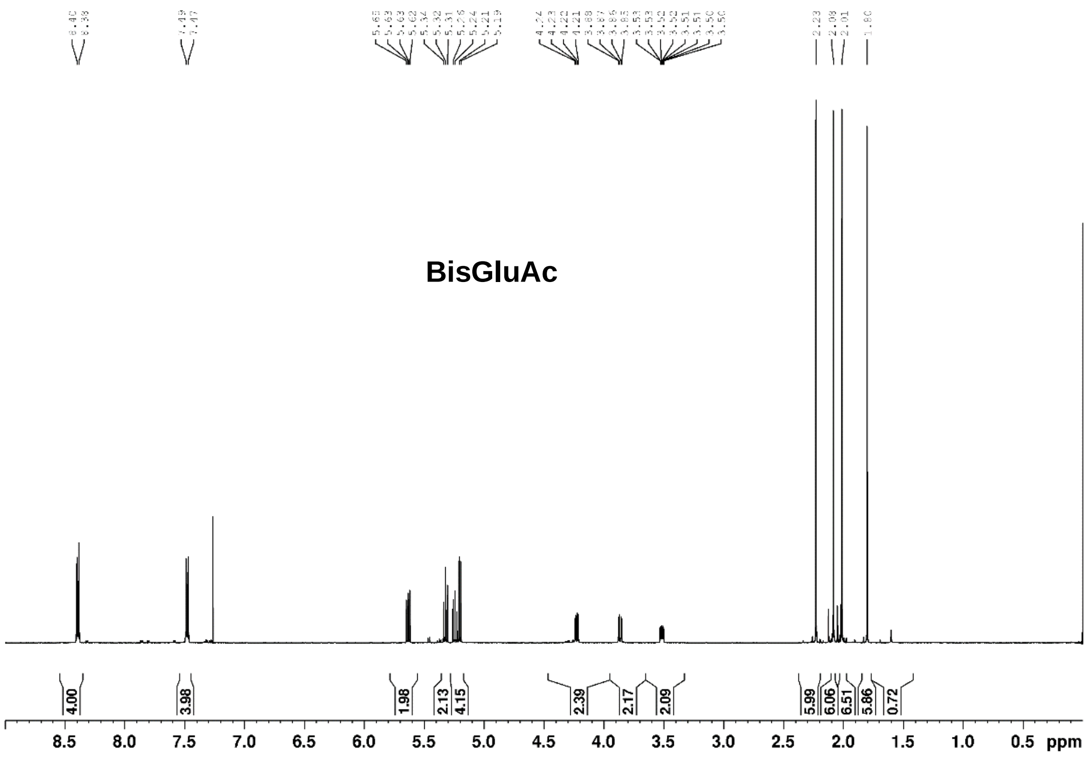

Figure S3

^{13}C NMR (125 MHz) spectrum

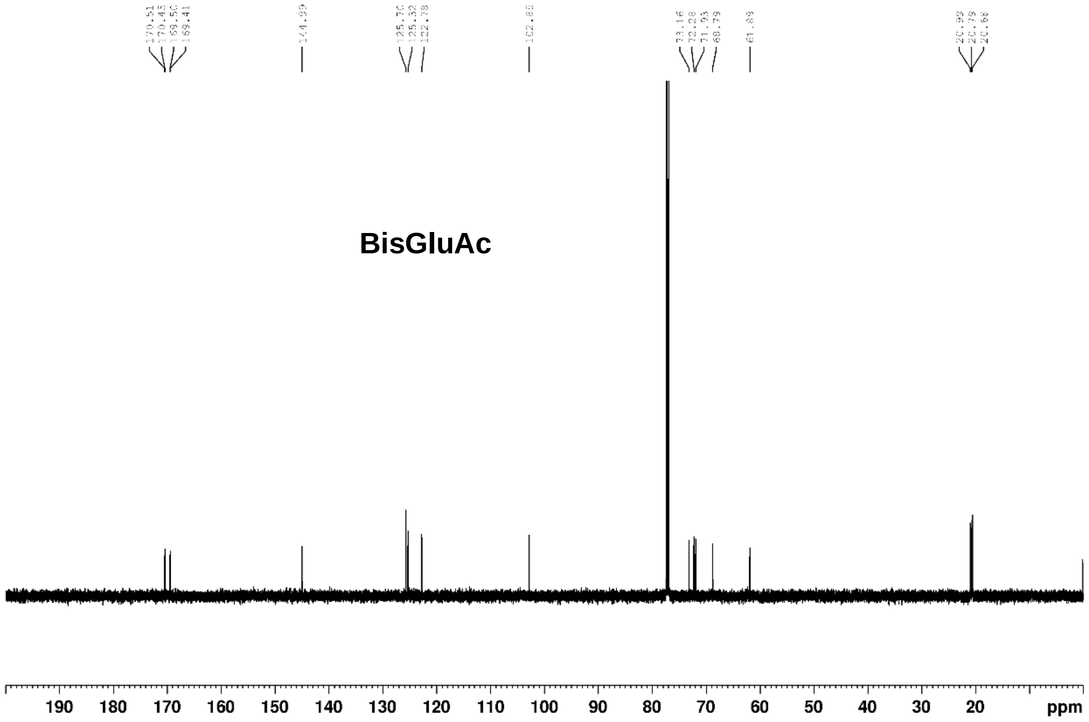

Figure S4

^{1}H NMR (500 MHz) spectrum

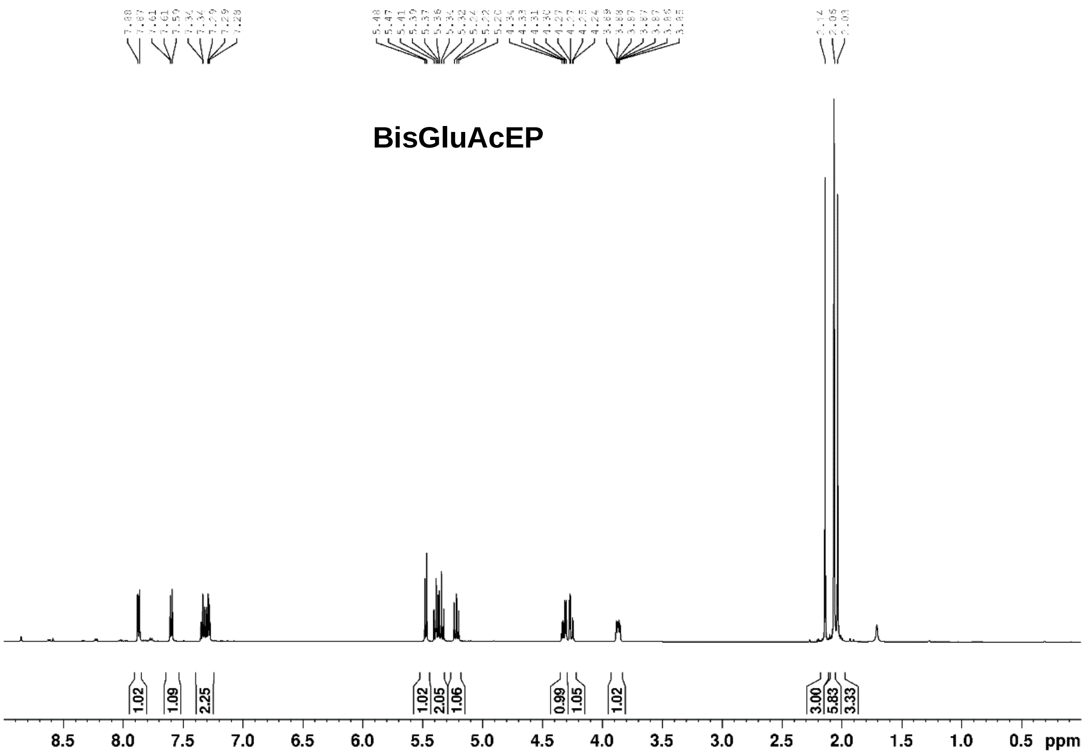

Figure S5

^{13}C NMR (125 MHz) spectrum

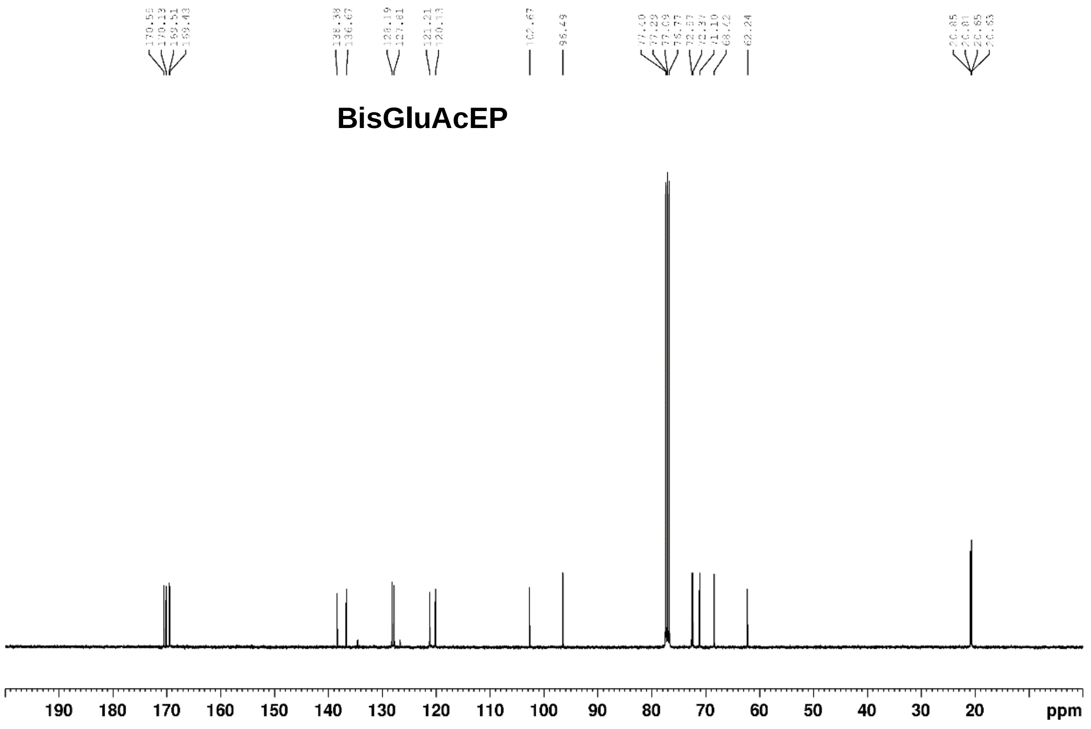

Figure S6

^{1}H NMR (500 MHz) spectrum

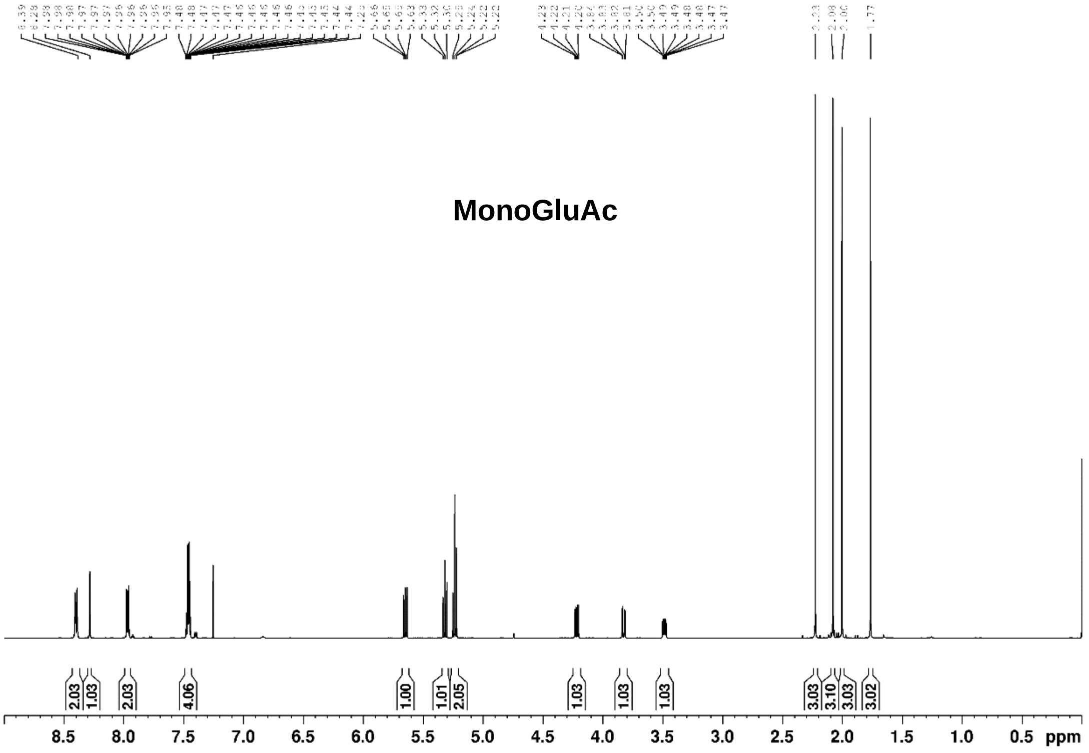

Figure S7

^{13}C NMR (125 MHz) spectrum

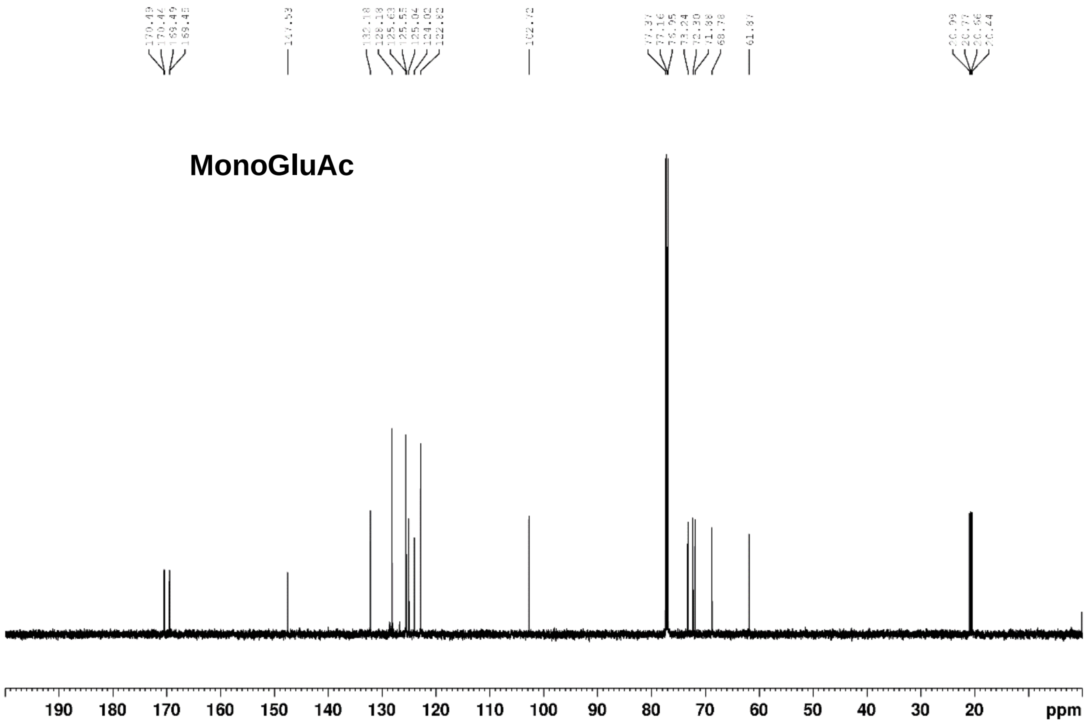

Figure S8

^{1}H NMR (500 MHz) spectrum

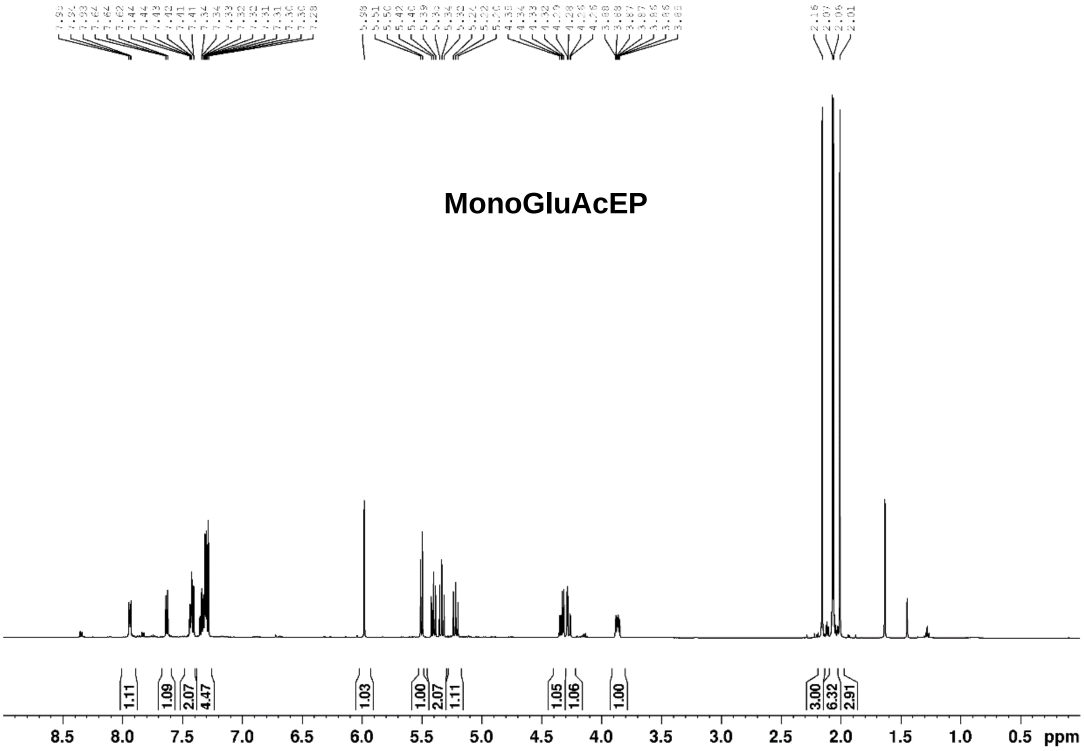

Figure S9

^{13}C NMR (125 MHz) spectrum

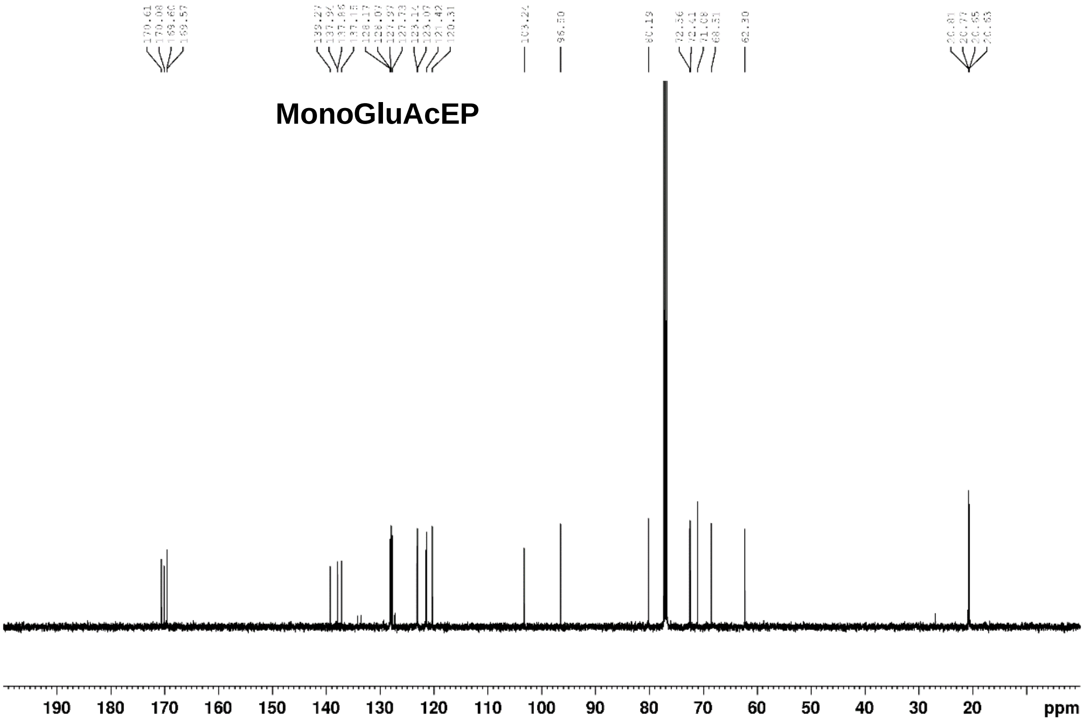

Figure S10

^{1}H NMR (400 MHz) spectrum

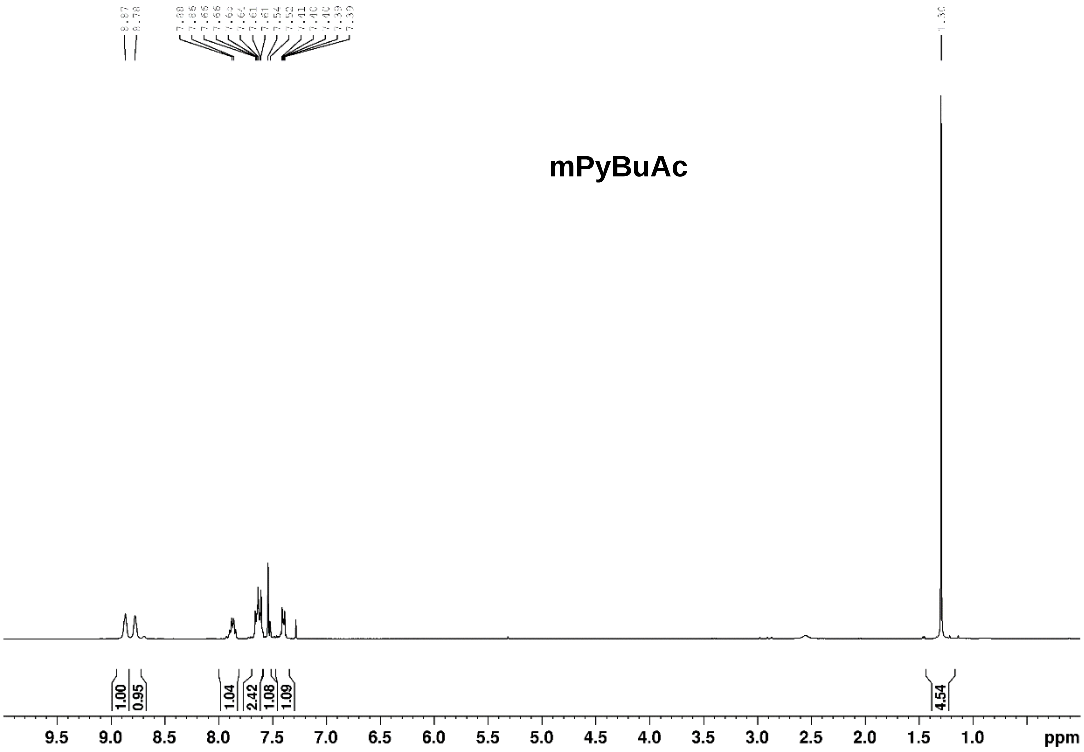

Figure S11

^{13}C NMR (100 MHz) spectrum

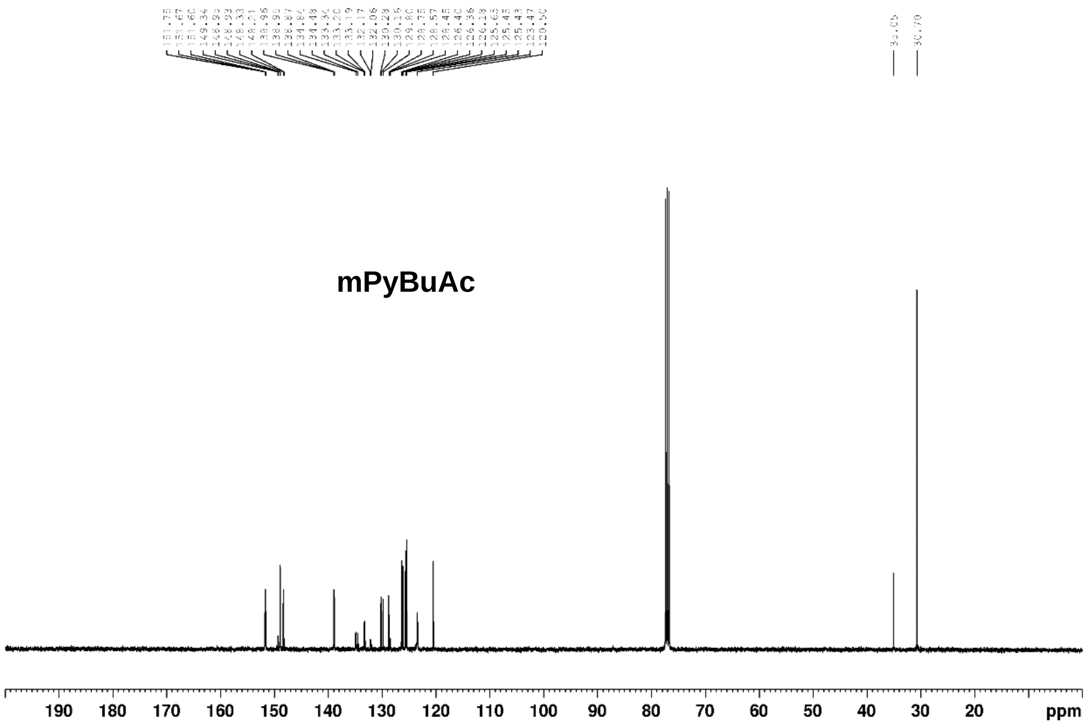

Figure S12

^{1}H NMR (400 MHz) spectrum

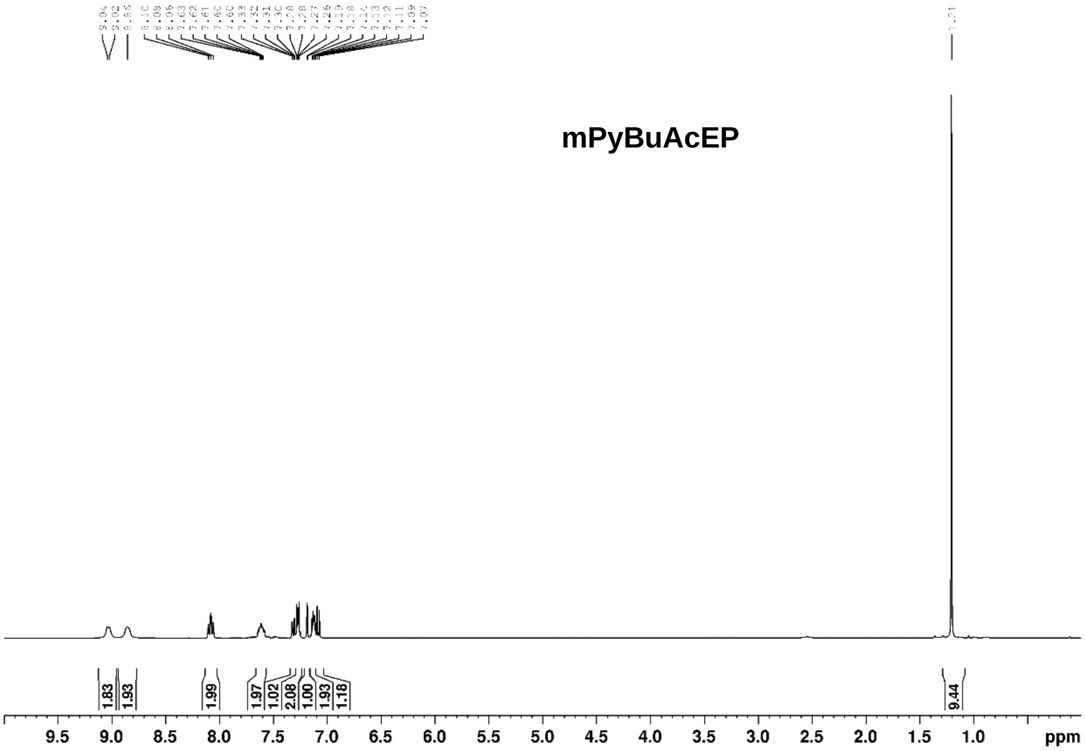

Figure S13

^{13}C NMR (100 MHz) spectrum

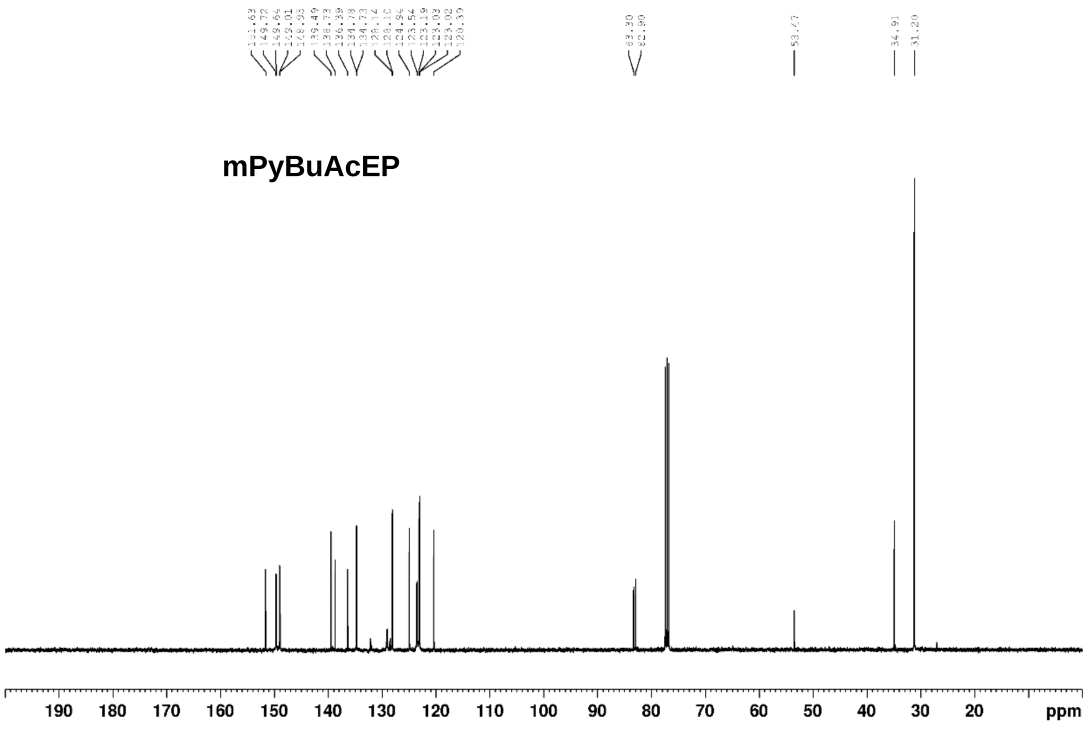

Figure S14

^{1}H NMR (400 MHz) spectrum

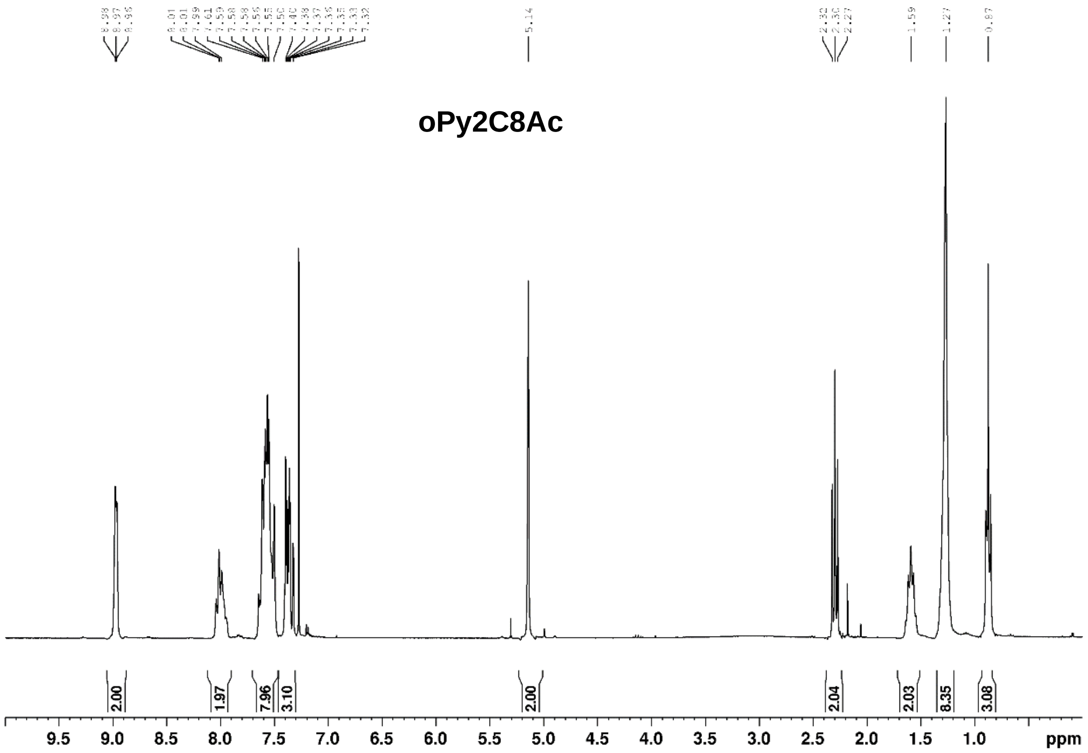

Figure S15

^{13}C NMR (100 MHz) spectrum

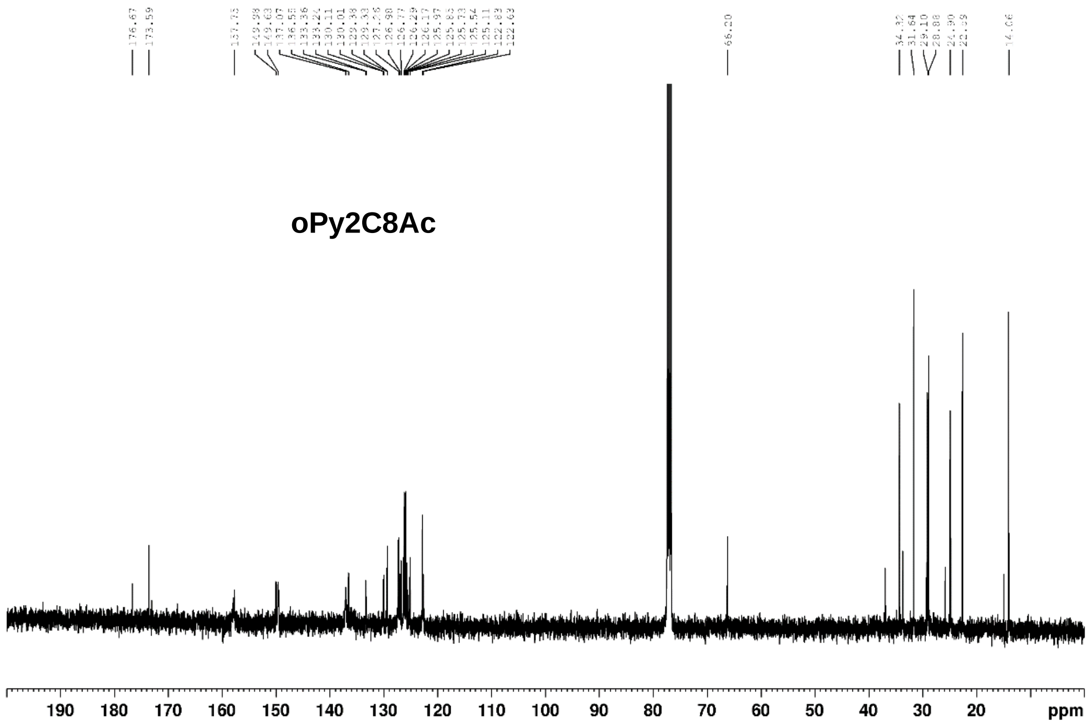

Figure S16

^{1}H NMR (400 MHz) spectrum

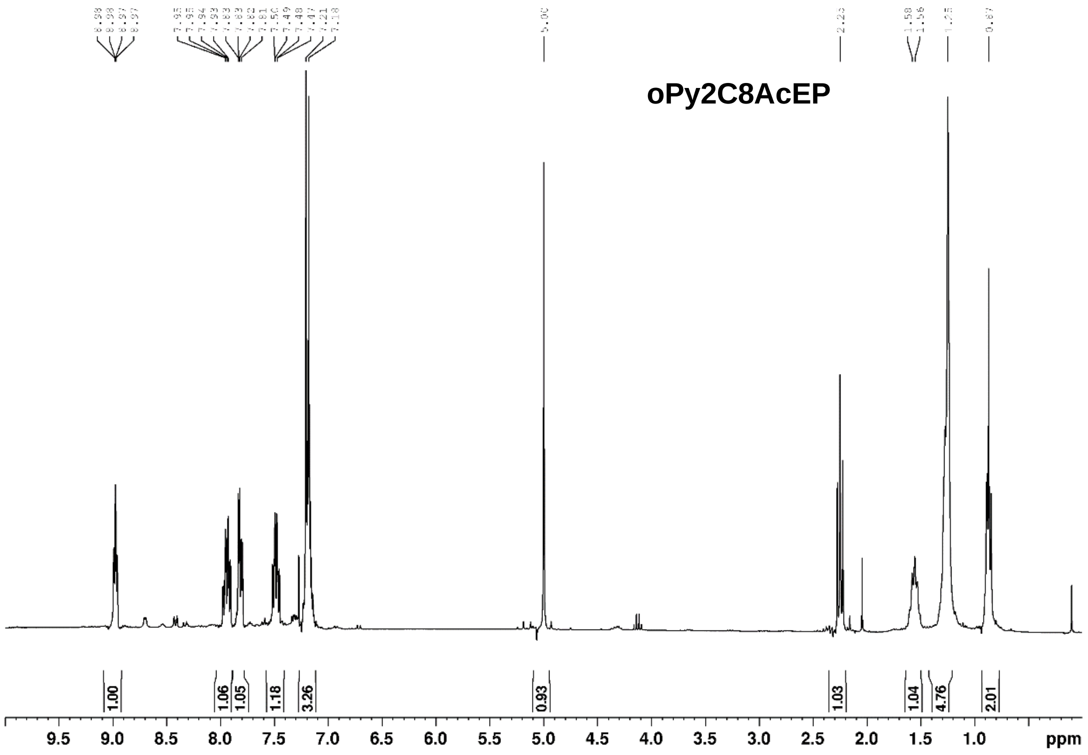

Figure S17

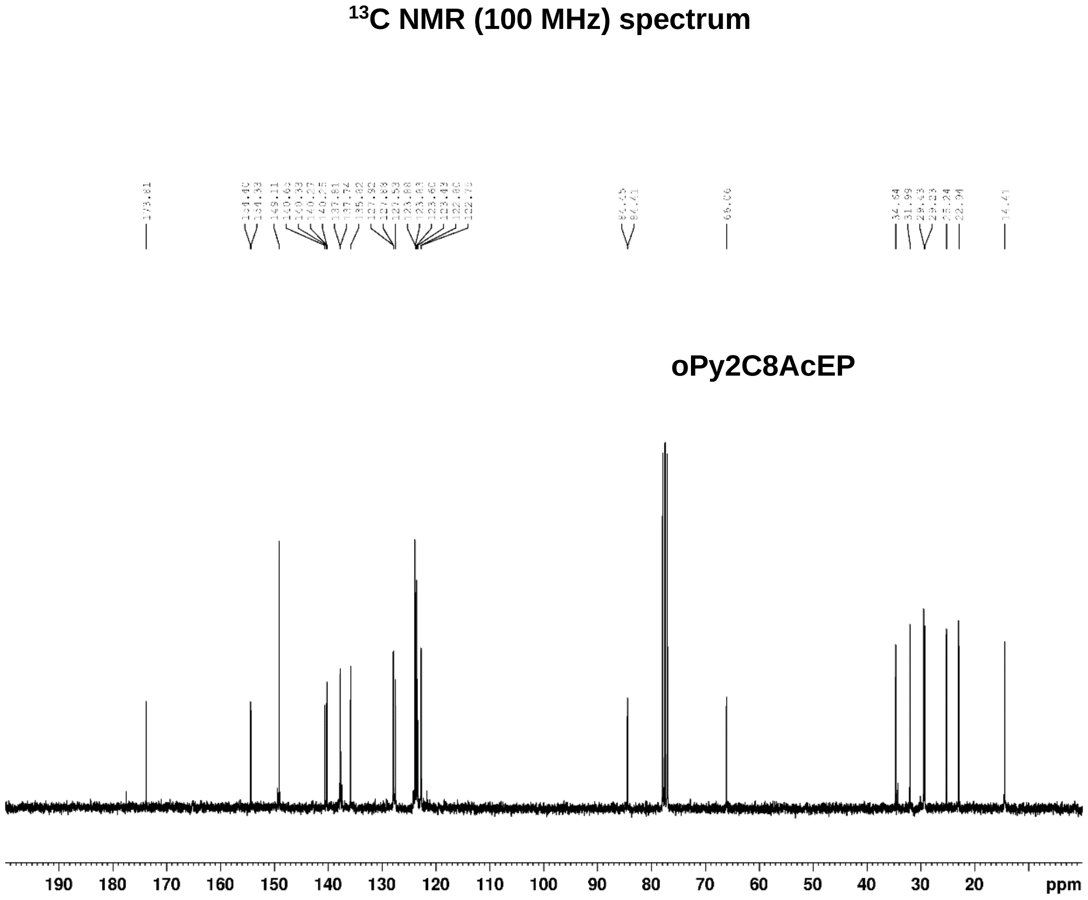

13C NMR (100 MHz) spectrum
oPy2C8AcEP
190
180
170
160
150
140
130
120
110
100
90
80
70
60
50
40
30
20
ppm
